# LncRNA EPB41L4A-AS1 regulates osteogenic differentiation and bone microenvironment-associated functions in ADSCs by targeting miR-302d-3p

**DOI:** 10.1186/s41065-026-00721-7

**Published:** 2026-08-11

**Authors:** Yiwei Wang, Xintong Hao, Enxiong Liu, Hui Peng

**Affiliations:** 1 Department of General Orthopedics, The First Hospital of Qinhuangdao, Qinhuangdao, 066000 China; 2 Department of Pain Medicine, Wuhan No.1 Hospital, Wuhan, 430022 China; 3 Department of Orthopedics, Fufeng People’s Hospital, Baoji, 722299 China; 4 Department of Orthopedics, Minzu Hospital of Guangxi Zhuang Autonomous Region, No. 232 Mingxiu East Road, Xixiangtang District, Nanning, 530001 China

**Keywords:** LncRNA, Adipose-derived mesenchymal stem cells, ceRNA, Osteoblast differentiation

## Abstract

**Background:**

Postmenopausal osteoporosis (PMOP) is a prevalent female bone disease. It can progress to fractures at advanced stages and raise disability risks.

**Objectives:**

This study evaluated the value of EPB41L4A-AS1 in PMOP diagnostic and fracture risk prediction, and explored its mechanism of regulating osteogenic differentiation in an in vitro ADSC model.

**Methods:**

A total of 118 healthy controls and 146 PMOP patients (75 non-fracture and 71 fracture) were enrolled. Serum levels of EPB41L4A-AS1, miR-302d-3p, and CUL3 were detected. ROC curves and logistic regression were performed to assess its clinic value. The regulatory mechanisms of EPB41L4A-AS1, miR-302d-3p and CUL3 were investigated using adipose-derived mesenchymal stem cells (ADSCs) models and a series of cellular experiments.

**Results:**

Serum EPB41L4A-AS1 were downregulated in PMOP patients and further decreased in the fracture subgroup (*P* < 0.001). EPB41L4A-AS1 exhibited high diagnostic performance for PMOP (AUC = 0.818). Its expression was strongly correlated with key bone metabolism indicators. Low EPB41L4A-AS1 expression was an independent risk factor for PMOP (OR = 0.164) and fractures occurrence (OR = 0.208). During osteogenic induction of ADSCs, silencing EPB41L4A-AS1 significantly reduced osteogenic markers expression and mineralized nodule formation, and disrupted cellular function and bone microenvironment homeostasis. Mechanically, EPB41L4A-AS1 targeted miR-302d-3p, which further regulated CUL3. Rescue experiments confirmed that the EPB41L4A-AS1/miR-302d-3p/CUL3 axis modulated ADSC osteogenic differentiation and bone microenvironment homeostasis.

**Conclusion:**

EPB41L4A-AS1 serves as a marker for diagnosis and fracture risk prediction in PMOP patients, and in vitro evidence suggests it regulates ADSC osteogenic differentiation via miR-302d-3p/CUL3, providing an in vitro mechanistic proof-of-concept.

**Supplementary Information:**

The online version contains supplementary material available at https://doi.org/10.1186/s41065-026-00721-7.

## Introduction

Postmenopausal osteoporosis (PMOP) is a common age-related bone metabolic disorder in women. It frequently leads to postmenopausal osteoporotic fractures (PMOF) in the middle and late disease stages, which greatly elevates the risk of disability and mortality [1]. Clinically, bone mineral density (BMD) measurement and traditional bone metabolism markers are routinely used to assist diagnosis and treatment. However, these approaches have insufficient sensitivity for detecting early lesions, hindering timely intervention and precise molecular targeted treatment [2]. Therefore, screening specific biomarkers for PMOP and exploring their regulatory mechanisms are of great significance for optimizing clinical strategies.

Long non-coding RNAs (lncRNAs) play critical roles in regulating stem cell function and maintaining bone metabolic homeostasis [3]. EPB41L4A-AS1, a well-characterized lncRNA, participates in a variety of molecular regulatory processes [4]. In osteosarcoma, high EPB41L4A-AS1 expression drives malignant progression and acts as a potential diagnostic biomarker [5]. It also facilitates osteogenic differentiation and alleviates inflammatory damage in LPS-induced periodontal fibroblasts [6]. However, periodontal fibroblasts primarily maintain local periodontal tissue integrity and possess limited multilineage differentiation potential compared with mesenchymal stem cells (MSCs). Overexpression of EPB41L4A-AS1 in bone marrow mesenchymal stem cells (BMSCs) significantly promotes cell proliferation [7], which directly proves its regulatory effect on adult stem cell function. While BMSCs are the principal osteoprogenitors in vivo, ADSCs provide an attractive alternative for PMOP research: they are more accessible, highly proliferative, and possess comparable osteogenic potential [8], while their systemic dysfunction in PMOP—where adipose tissue is significantly altered—directly contributes to impaired bone metabolism [9]. The specific regulatory role and clinical translational value of EPB41L4A-AS1 and downstream ceRNA mechanism in ADSC osteogenesis remain unexplored. Moreover, EPB41L4A-AS1 has been reported as a therapeutic target for aging and age-related diseases including Alzheimer’s disease and type 2 diabetes, as it regulates metabolic and inflammatory processes [10]. As a typical age-related disease, PMOP shares similar pathological characteristics with the above disorders. Nevertheless, whether EPB41L4A-AS1 specifically regulates ADSC osteogenic differentiation and bone microenvironment homeostasis in the context of PMOP remains unknown, representing a critical gap that this study aims to address.

Accumulating evidence indicates that microRNAs (miRNAs) regulate the development of bone-related diseases by modulating osteoblast differentiation, chondrocyte function, angiogenesis, and apoptosis [11]. miR-302d-3p is a key regulator of cell differentiation, inflammation, and extracellular matrix metabolism. In osteoarthritis (OA), miR-302d-3p accelerates chondrocyte apoptosis and matrix degradation [12], and mediates inflammatory responses through the NF-κB or PI3K-AKT signaling pathways [13, 14], thereby exacerbating OA progression. As a differentially expressed miRNA in intervertebral disc degeneration (IDD), miR-302d-3p also regulates immune responses in the lesion microenvironment [15]. Additionally, miR-302d-3p interacts with lncRNAs to form ceRNA axes in human adipose-derived stromal stem cells, regulating inflammatory signaling and adipogenic differentiation [16], which provides new insights into the fate determination of adult stem cell. Bioinformatics prediction reveals that EPB41L4A-AS1 has potential complementary binding sites with miR-302d-3p. However, it remains unknown whether the two molecules participate in the occurrence and progression of PMOP through targeted binding.

Given the above research gaps, this study assessed the diagnostic and predictive value of serum EPB41L4A-AS1 for PMOP. Using cellular models, we further explored the targeting interaction between EPB41L4A-AS1 and miR-302d-3p, as well as their regulatory effects on osteogenic differentiation and the bone microenvironment, aiming to elucidate the mechanistic basis of PMOP pathogenesis.

## Patients and methods

### Research subjects and basic information collection

A total of 264 postmenopausal women who received medical treatment or routine physical examination at Fufeng People’s Hospital between February 2021 and December 2023 were enrolled in this study. All participants had experienced natural menopause for no less than 12 months and were divided into two groups based on clinical assessments. All subjects signed written informed consent prior to enrollment. The control group consisted of 118 women with normal BMD, no history of osteoporosis-related fractures and no family history of osteoporosis. The PMOP group included 146 women whose BMD T-scores of the 1st to 4th lumbar vertebrae (L1–L4), femoral neck (FN) or total hip (TH) were ≤ − 2.5, meeting the diagnostic criteria for osteoporosis. According to the presence of fragility fractures within one year prior to enrollment (confirmed by imaging examinations), PMOP patients were further divided into the non-fracture subgroup (*n* = 75) and a fracture subgroup (*n* = 71).

Exclusion criteria were: (1) individuals with severe liver or renal dysfunction, malignant tumors, or autoimmune disorders that affect bone homeostasis; (2) those taking drugs interfering with bone turnover; (3) subjects with a history of severe infection, trauma, surgery, hemorrhagic diseases, mental disorders, or cognitive impairment within the past three months; (4) those who sustained fragility fractures within one year before enrollment.

After enrollment, clinical data including age, menopausal age, BMI, and medical history of hypertension, hyperlipidemia, diabetes, and previous fractures were collected. BMD measurements at the L1–L4, FN, and TH, along with corresponding T-scores, were obtained by certified professional technicians. The study protocol was reviewed and approved by the Medical Ethics Committee of our institution.

### Serum collection and laboratory index detection

Fasting venous blood was collected in the early morning. The blood was transferred into vacuum tubes and left to clot at room temperature for 30 min. Samples with visible hemolysis were strictly excluded from subsequent analysis. Following centrifugation at 4 °C and 3000 r/min for 15 min (Eppendorf, Germany; RRID: SCR_019848), the supernatant serum was carefully separated and immediately aliquoted to prevent repeated freeze-thaw cycles. Aliquots were stored at − 80 °C until RNA extraction, with only a single thaw performed prior to analysis.

Serum concentrations of 25-hydroxyvitamin D₃ [25(OH)D₃], β-CTX, OCN, and estradiol (E2) were measured using ELISA (R&D Systems, USA). Serum calcium ion (Ca²⁺) concentration was measured using a fully automated biochemical analyzer (Hitachi, Japan). All assays were performed in strict accordance with laboratory quality control standards.

### Cell culture and osteogenic induction

Human ADSCs (Cyanen, China; Catalog: HUXMD-01001) were commercially obtained from healthy female donors. The cells were isolated from subcutaneous white adipose tissue harvested via minimally invasive liposuction from the abdominal region. Donors were pre-menopausal women aged 32–48 years, with BMI strictly controlled within the normal physiological range of 18.5–23.9 kg/m². All donors were confirmed to be free of osteoporosis, endocrine disorders, and metabolic diseases through comprehensive pre-donation physical examinations. Donors provided informed consent, and cell production complied with ethical standards for human biological materials, with complete donor traceability and ethical documentation provided by the manufacturer. The use of female-derived ADSCs aligns with the sex-specific pathological background of PMOP investigated in this study, thereby minimizing inter-species and sex-based heterogeneity.

Cells were cultured in a ready-to-use specialized medium (Cyanen, China) containing fetal bovine serum, penicillin, streptomycin, and culture additives. Cells were incubated in a Sparkjade (China) incubator (37 °C and 5% CO₂). When the cell fusion degree reached 80%, the cells were digested and passed. Cells at passage 3 (P3) from cryopreserved stocks, confirmed by the manufacturer’s quality control report to possess qualified trilineage differentiation potential (adipogenic, osteogenic, and chondrogenic) and to express the characteristic mesenchymal stem cell surface markers (positive for CD73, CD90, and CD105; negative for CD34 and CD45) as assessed by flow cytometry, were selected for subsequent experiments to minimize experimental bias arising from inter-donor variability. All in vitro experiments were performed with 5 independent biological replicates and 3 technical replicates per biological sample.

ADSCs were seeded into 6-well plates (Corning, USA; Catalog: 3516) at a density of 2 × 10⁵ cells/well. At this stage, osteogenic differentiation was initiated using the OriCell^®^ protocol for the human adipose-derived mesenchymal stem cell osteogenic induction kit (Cyanen, China; Catalog: GUXMX-90041) to establish in vitro osteogenic differentiation model. Cells and culture supernatants were harvested at designated time points—day 0, 7, 14, and 21—for subsequent analysis.

### Cell transfection

The sequences of oligonucleotides used in this study were as follows: si-EPB41L4A-AS1, 5′-GGATGTCCTTGGTGAGGATTT-3′; si-CUL3, 5′-GCACAUGAAGACUAUAGUA-3′; mimic-miR-302d-3p, 5′-UAAGUGCUUCCAUGUUUGAGUGU-3′; inhibitor-miR-302d-3p, 5′-ACACUCAAACAUGGAAGCACUUA-3′; and their corresponding negative controls [si-NC, 5′-TTCTCCGAACGTGTCACGT-3′; si-NC (CUL3), 5′-UGGUUUACAUGUCGACUAA-3′; mimic-NC, 5′-UUCUCCGAACGUGUCACGUTT-3′; inhibitor-NC, 5′-CAGUACUUUUGUGUAGUACAA-3′]. All oligonucleotides were chemically synthesized by Obiosh Corporation (China) as indicated. One day before cell transfection, osteogenesis-induced ADSCs were seeded onto a 6-well plate at a density of 2 × 10⁵ cells/well. The PEI MAX transfection reagent (MW40000, Polysciences, USA; Catalog: 24765) was used for transfection at a final concentration of 1 µg/mL. The siRNA or miRNA mimic/inhibitor was complexed with PEI MAX at a nitrogen-to-phosphate (N/P) ratio of 10:1 in serum-free Opti-MEM medium (Gibco, USA; Catalog: 31985070) for 15 min at room temperature before addition to cells. After 24 h, the medium was replaced and cultured for a further 48 h before the cells and supernatant were collected. Each transfection experiment was performed with 5 independent biological replicates, and each sample was analyzed in 3 technical replicates.

### Fluorescence quantitative PCR (qPCR) detection

Total RNA was isolated from serum samples and cells using the MolPure^®^ Serum/Plasma or Cell/Tissue RNA Kit (Yeasen, China; Catalog: 19332ES50). RNA purity and concentration were assessed using a Nanodrop spectrophotometer, and only samples with an A260/A280 ratio of 1.8–2.0 and an A260/A230 ratio > 1.5 were included for downstream analysis.

For cDNA synthesis, one microgram of total RNA was reverse transcribed using either the miRNA 1st Strand cDNA Synthesis Kit (Arcegen, USA; Catalog: AG11728) or the Fast 1st Strand cDNA Synthesis Kit (Arcegen, USA; Catalog: AG11706).

Quantitative PCR was performed using the SYBR Green Pro Taq HS Premixed qPCR Kit (Agbio, China; Catalog: AG11701) on the SmartCycler^®^ System (Cepheid, USA), following the recommended reaction conditions and cycling parameters outlined in the product manuals. GAPDH/U6 served as the internal control for gene expression (using the 2^−ΔΔCt^ method). In addition to osteogenic marker genes, qPCR was also used to assess the mRNA expression levels of pro-angiogenic factors (VEGF, bFGF, ANG-1, and PDGF) in transfected ADSCs. The primers were obtained from Beijing RuiBo XingKe Biotechnology Co., Ltd., are detailed in Supplementary Table 1. Each qPCR assay was conducted using 5 independent biological replicates, with each reaction run in 3 technical replicates.

For serum lncRNA and mRNA quantification, GAPDH was employed as the internal reference gene, while U6 was used for miRNA normalization. The selection of GAPDH as a housekeeping control for circulating lncRNA/mRNA analysis was based on its extensive validation and widespread application in prior serum biomarker studies [17, 18]. GAPDH has been confirmed to exhibit consistent and stable expression in serum samples from both healthy controls and patient cohorts, and has been specifically validated as a reliable internal control for lncRNA normalization in circulation [19]. To address the concern that GAPDH stability in serum may be affected by hemolysis or exosome release dynamics, we additionally compared the raw Ct values of serum GAPDH across the three study groups (healthy control, PMOP non-fracture, and PMOP fracture). One-way ANOVA revealed no statistically significant difference in GAPDH Ct values among the three groups (F = 0.256, *P* = 0.774), indicating stable expression of this endogenous reference across cohorts (Supplementary Fig. 3; Supplementary File 1). Notably, exogenous spike-in controls were not employed in this study. While such exogenous controls can effectively monitor technical variations during RNA extraction and reverse transcription, they cannot account for inherent biological differences in circulating RNA concentrations across samples (e.g., hemolysis or hydration status). Therefore, this study relied on stringent preanalytical quality control—including strict exclusion of hemolyzed samples, single-thaw processing, and standardized extraction protocols—coupled with validated endogenous reference genes (GAPDH for lncRNA/mRNA and U6 for miRNA) and sufficient biological and technical replicates to ensure the reliability of circulating RNA quantification.

### Nuclear-cytoplasmic separation

ADSCs were grown until they reached more than 80% confluence. The cells were then detached by treating them with 0.25% trypsin at 37 °C for 2–3 min. Digestion was stopped by adding the appropriate culture medium, and the resulting cell suspension was transferred into an RNase-free centrifuge tube. Cells were collected by centrifugation at 300 × g for 5 min at room temperature. The supernatant containing the culture medium was carefully discarded, and the cell pellet was washed 2–3 times with ice-cold PBS to completely remove any remaining medium. Next, cytoplasmic and nuclear fractions were isolated strictly following the manufacturer’s protocol for the PARIS™ Kit (Ambion, USA). Total RNA was separately extracted from both fractions. RNA purity was measured using a Nanodrop spectrophotometer. High-quality RNA samples were then reverse transcribed into cDNA using a reverse transcription kit, and qPCR was performed to assess the relative expression levels of EPB41L4A-AS1 in the cytoplasmic and nuclear fractions. U6 was used as the internal control for the nuclear fraction, while GAPDH served as the reference gene for the cytoplasmic fraction, allowing normalization of RNA recovery efficiency and correction for experimental variation between subcellular fractions.

### RNA immunoprecipitation (RIP) experiment

ADSCs in the logarithmic growth phase were collected and analyzed using RIP assays with a RIP assay kit (Millipore, USA) to examine the interaction of AGO2 with EPB41L4A-AS1, miR-302d-3p, and CUL3. Cells were lysed on ice at 4 °C for 5 min using RIPA lysis buffer (Beyotime, China), then centrifuged at 14,000 rpm for 10 min. 10% of the lysate was set aside as the input control, while the rest was used for co-immunoprecipitation. Immune complexes were generated by incubating the supernatant overnight at 4 °C with AGO2 antibody bound to Protein A/G magnetic beads (5 µg, diluted 1:50, Abcam, UK) or with normal rabbit IgG antibody as a negative control (5 µg, diluted 1:100, Abcam, UK). After washing with 900 µL of RIP washing buffer and resuspension, 100 µL of cell lysate was added and incubated overnight at 4 °C. Following incubation, samples were washed, and the magnetic bead-protein complexes were collected. Both the immunoprecipitated complexes and input samples were treated with proteinase K (Beyotime, China) to digest proteins, followed by RNA extraction. The levels of EPB41L4A-AS1, miR-302d-3p, and CUL3 enrichment were then measured by qPCR.

### Western blot (WB) assay

Following transfection, the ADSCs were added to RIPA lysis buffer (Epizyme, China; Catalog: P0013B) supplemented with 1% PMSF (Beyotime, China; Catalog: ST506). After 30 min of ice lysis, the sample was centrifuged at 12,000 × g for 15 min at 4 °C. The resulting supernatant was determined using a BCA protein assay kit (Solarbio, China; Catalog: P0010). The denatured protein was separated by electrophoresis using a 10% SDS-PAGE gel (GenStar, China; Catalog: M00677). Subsequently, the MiniBlot™ protein transfer system (Beyotime, China; Catalog: E6080) was used to transfer proteins to PVDF membranes (Epizyme, China; Catalog: IPVH00010) at 100 V for 60 min.

After transfer printing, the membrane was sealed for 10 min using Fast Blocking Western (Yeasen, China; Catalog: 36122ES76) at room temperature. After washing with TBST (Beyotime, China; Catalog: ST673) three times (5 min each), the following primary antibodies were added and incubated overnight at 4 °C: MMP-2 antibody (1:500; SCBT, USA; RRID: AB_2250826), TIMP-1 antibody (1:500; SCBT, USA; RRID: AB_628359), Nrf2 antibody (1:500; Abcam, UK; RRID: AB_137550) and GAPDH antibody (1:1000; Beyotime, China; RRID: AB_3740809). The next day, after washing the membrane three times with TBST (10 min each), HRP-labelled secondary antibody (1:5000; Starter-Bio, China; Catalog: A0216/A0208) was incubated for 1 h. An ultra-sensitive ECL chemiluminescence assay kit (NCM Biotech, China; Catalog: P0018FS) was used with a BeyoImager™ 600 chemiluminescence imaging system (Beyotime, China) for development. Protein band intensities were quantified using ImageJ software (NIH, USA; RRID: SCR_003070). Western blot analysis was performed on 5 independent biological replicates, and each sample was loaded in 3 technical replicates.

### Detection of alkaline phosphatase (ALP) activity

ADSCs undergoing osteogenic induction were harvested at days 0, 7, 14, and 21, along with transfected ADSCs. Cells were lysed in RIPA buffer and incubating on ice for 30 min. Following centrifugation at 12,000 × g for 15 min at 4 °C, the supernatant was collected for analysis.

For ALP activity assessment, 50 µL of cell lysate was mixed with 50 µL of substrate solution (ALP Detection Kit, Dakewe, China; Catalog: DKW12-K030) and incubated at 37 °C for 30 min. Absorbance (OD value; 405 nm) was measured using a Varioskan LUX microplate reader (Thermo Fisher, USA; Catalog: VL0000D0), and ALP activity was determined by interpolation from a standard curve. ALP activity was measured in 5 independent biological replicates, with each sample analyzed in 3 technical replicates.

### Alizarin red S (ARS) staining analysis

The ADSCs that had undergone osteogenic differentiation induction and cell transfection were washed with PBS and fixed with 4% paraformaldehyde (Beyotime, China; Catalog: P0099) at room temperature for 30 min. After rinsing the cells three times with deionized water, a 40 mM ARS staining solution (Sigma-Aldrich, USA; Catalog: A5533) was added and incubated for 20 min at room temperature. The cells were slowly washed with distilled water until the background was colorless, after which they were inverted and dried. One milliliter of a 10% acetic acid solution (Solis BioDyne, USA) was added and the plate was incubated for 30 min with gentle shaking. Then, 200 µL of the solution was transferred to a 96-well plate. The OD value (415 nm) was then measured using an ELISA reader (Thermo Fisher, USA; Catalog: VL0000D0). ARS staining was performed on 5 independent biological replicates, and the OD value was measured in 3 technical replicates per sample.

### Cell proliferation, apoptosis and migration assays

After transfection, ADSCs were seeded into a 96-well plate at a density of 3 × 10³ cells/well. At 0, 24, 48, 72, and 96 h post-seeding, 10 µL of CCK-8 solution (Fine Test, China; Catalog: IF9164) was added to each well. Following a 2-hour incubation at 37 °C, absorbance (450 nm) was measured using an ELISA reader (Thermo Fisher, USA; Catalog: VL0000D0). The recorded OD values reflected cellular proliferation activity, and a growth curve was generated accordingly. Cell proliferation assays were conducted with 5 independent biological replicates, and each time point was measured in 3 technical replicates.

For apoptosis analysis, transfected cells were harvested by trypsinization. The cell suspension was adjusted to a density of 1 × 10⁶ cells/mL. According to the protocol (Annexin V-FITC/PI Apoptosis Detection Kit, BD, USA; RRID: AB_2869082), 5 µL of Annexin V-FITC and 5 µL of propidium iodide were sequentially added to 100 µL of cell suspension, followed by a 15-minute incubation in the dark at room temperature. After adding 400 µL of binding buffer, samples were analyzed using a BD FACSCanto II flow cytometer, and data were processed with FlowJo software (BD Biosciences, USA; RRID: SCR_008520). Apoptosis assays were performed on 5 independent biological replicates, and each sample was analyzed in 3 technical replicates.

To assess cell migration, transfected ADSCs were resuspended in medium (serum-free). A volume of 200 µL (containing 5 × 10⁴ cells) was placed in the upper compartment of a Transwell insert (8.0 μm pore size, Corning, USA; Catalog: 3422), while 600 µL of culture medium containing 10% FBS was added to the lower chamber. Cells were incubated for 24 h under standard condition (37 °C, 5% CO₂). Subsequently, cells on the upper side of the membrane were removed with a cotton swab. Migrated cells were fixed with 4% paraformaldehyde for 20 min, stained with 0.1% crystal violet (Solarbio, China; Catalog: G1063) for 15 min, rinsed three times with PBS, and air-dried. Cell migration was quantified by counting migrated cells using a Leica microscope (Germany). Migration assays were performed with 5 independent biological replicates, and migrated cells were counted in 3 randomly selected fields per insert (technical replicates).

### Dual-luciferase reporter gene assay

The potential binding site between EPB41L4A-AS1 and miR-302d-3p, as well as miR-302d-3p and the 3′-UTR of CUL3 were predicted using the bioinformatics tool StarBase (http://starbase.sysu.edu.cn/). Wild-type (WT) and mutant (MUT) luciferase reporter vectors were generated by GenScript (China), where the WT construct contained the original binding sequence and the MUT version carried a modified seed region. Both constructs were cloned into the pmirGLO vector backbone (Promega, USA; Catalog: E1330).

ADSCs were seeded into 96-well plates at a density of 1 × 10⁴ cells/well and cultured for 24 h prior to transfection. The WT or MUT vectors were co-transfected into cells using PEI MAX (MW40000) as the transfection reagent, along with either mimic-miR-302d-3p (50 nM), inhibitor-miR-302d-3p (100 nM), or their respective NCs. After 48 h, dual-luciferase activity was assessed using the Firefly & Renila Assay Kit (Absin, China; Catalog: abs60341) following the manufacturer’s protocol. Luminescence signals were measured with a multifunctional microplate reader (Thermo Fisher, USA; Catalog: VL0000D0) capable of detecting both firefly and Renila luciferase. Relative luciferase activity was determined by normalizing firefly values to Renila readings. The dual-luciferase reporter assay was performed using 5 independent biological replicates, and luciferase activity was measured in 3 technical replicates for each sample.

### Detection of inflammatory factors

Following transfection, the ADSC culture supernatant was collected and centrifuged at 4 °C and 3000 r/min for 15 min to remove cellular debris. The resulting supernatant was used to quantify levels of TNF-α, IL-6, IL-10, and TGF-β1 with an ELISA kit (Coibo Bio, China; Catalog: CK-E30649, CK-E30613, CK-E30647, CK-E30656, respectively). All steps, including antibody incubation, binding reactions, and substrate development, were carried out under specified conditions as outlined in the instructions. Absorbance (450 nm) was measured using an ELISA microplate reader, and cytokine concentrations were determined by interpolation from standard curves. Relative levels of each inflammatory factor were then calculated accordingly. Inflammatory factor detection was performed on 5 independent biological replicates, and each cytokine was measured in 3 technical replicates.

### Target gene screening

Potential target genes of miR-302d-3p were predicted through computational analysis using several public databases, such as miRDB, TargetScan, and miRTarBase. Genes that appeared in all three databases were chosen for further investigation. Next, a protein–protein interaction (PPI) network was constructed using the STRING platform, and the six genes with the highest connectivity (i.e., greatest degree centrality) were identified as hub genes.

### Statistical analysis

Continuous variables were first assessed for normality using the Shapiro–Wilk test and for homogeneity of variance using Levene’s test. The continuous variable satisfied both conditions simultaneously. The t-test was used to compare continuous variables between two groups and analysis of variance was used to compare multiple groups, followed by a Bonferroni correction (for planned pairwise comparisons) to control the family‑wise error rate. Categorical variables were compared using the chi‑square (χ²) test (expected cell frequency ≥ 5) or Fisher’s exact test when expected cell frequencies were < 5. Bivariate associations were evaluated using Pearson (for normally distributed data) or Spearman rank (for skewed data) correlation coefficients. A multivariate binary logistic regression model was used to analyze the association between various clinical indicators and the occurrence of PMOP, as well as the risk of fracture. ROC curve analysis was used to evaluate the diagnostic value of EPB41L4A-AS1 for PMOP. All statistical analyses were performed using SPSS 26.0 (IBM, USA; RRID: SCR_002865) and GraphPad Prism 9.0 (GraphPad, USA; RRID: SCR_002798). A two-tailed *P*-value < 0.05 was considered statistically significant.

## Results

### Baseline and laboratory characteristics of the study subjects

Sample size determination (GPower 3.1) was conducted a priori using two-tailed independent-samples t-tests, with an effect size (Cohen’s d) of 0.5, significance level α = 0.05, and desired statistical power (1 − β) of 0.80. This yielded a minimum required sample size of 67 participants per group. The final cohort comprised 118 controls and 146 PMOP patients—both exceeding the calculated threshold. Post hoc power analysis confirmed high achieved power: 0.97 for the control versus PMOP comparison, and 0.83 for the PMOP subgroup analysis (75 non-fracture vs. 71 fracture cases), both meeting or exceeding the prespecified power target.

There were no significant differences in age, menopausal age, or BMI between groups (*P* > 0.05), indicating balanced baseline demographic data. The incidence of hypertension, hyperlipidemia, and diabetes was slightly higher in the PMOP group, but the difference was not statistically significant (*P* > 0.05). The proportion of patients with previous fractures was markedly elevated in the PMOP group, reaching 25.35% in the fracture subgroup (*P* = 0.035).

Compared with the control group, BMD values at the L1–L4, FN, and TH, along with their corresponding T-scores, were significantly reduced in the PMOP group (*P* < 0.001), with further reductions in the fracture subgroup. Serum detection results showed that compared with the control group, the PMOP group had lower levels of 25(OH)D₃ (*P* = 0.017), Ca²⁺ (*P* = 0.041), and E2 (*P* < 0.001), while the levels of bone resorption markers β-CTX (*P* < 0.001) and OCN were significantly increased (*P* < 0.001). These above changes were more obvious in patients with fractures (Table 1).

**Table 1:** Clinical and laboratory characteristics of the patients

Parameters	Control (*N* = 118)	PMOP (*N* = 146)		*P* value
		Non-fracture (*n* = 75)	Fracture (*n* = 71)	
Age, year	56.32 ± 5.16	58.07 ± 6.20	57.27 ± 6.62	0.128
Age of menopause, year	49.12 ± 3.61	48.59 ± 2.58	48.06 ± 3.48	0.100
BMI, kg/m^2^	23.28 ± 2.85	23.91 ± 3.51	23.23 ± 2.91	0.301
Hypertension history (yes, %)	35 (29.66)	28 (37.33)	32 (45.07)	0.098
Hyperlipoidemia history (yes, %)	27 (22.88)	22 (29.33)	25 (35.21)	0.180
Diabetes history (yes, %)	19 (16.10)	16 (21.33)	21 (29.58)	0.090
Fracture history (yes, %)	13 (11.01)	12 (16.00)	18 (25.35)	**0.035**
L1-4 BMD, g/cm^2^	1.08 ± 0.11	0.88 ± 0.10	0.72 ± 0.10	**< 0.001**
FN BMD, g/cm^2^	0.92 ± 0.07	0.76 ± 0.10	0.66 ± 0.08	**< 0.001**
TH BMD, g/cm^2^	1.00 ± 0.09	0.82 ± 0.08	0.71 ± 0.09	**< 0.001**
T scores	-0.17 ± 0.58	-1.79 ± 0.38	-3.06 ± 0.43	**< 0.001**
25(OH)D_3_, ng/mL	24.21 ± 5.89	23.45 ± 6.19	21.62 ± 5.92	**0.017**
Ca^2+^, mmol/L	2.33 ± 0.15	2.31 ± 0.14	2.28 ± 0.13	**0.041**
β-CTX, ng/mL	0.38 ± 0.13	0.59 ± 0.16	0.68 ± 0.17	**< 0.001**
OCN, ng/mL	19.33 ± 5.03	22.36 ± 6.02	23.10 ± 6.78	**< 0.001**
E2, pmol/L	70.43 ± 21.30	30.20 ± 7.80	28.25 ± 6.32	**< 0.001**

*Abbreviations* *PMOP* postmenopausal osteoporosis, *BMI* body mass index, *L1-4 BMD* lumbar spine bone mineral density, *FN BMD * femoral neck bone mineral density, *TH BMD* total hip bone mineral density, *25(OH)D*_3_ 25-hydroxyvitamin D_3_, *Ca*^2+^ calcium ion, *β-CTX * β-isomerized C-terminal telopeptide of type I collagen, *OCN * osteocalcin, *E2 * Estradiol

Bold formatting denotes statistically significant differences

### Serum EPB41L4A-AS1 expression level and diagnostic efficacy

**Fig. 1:**
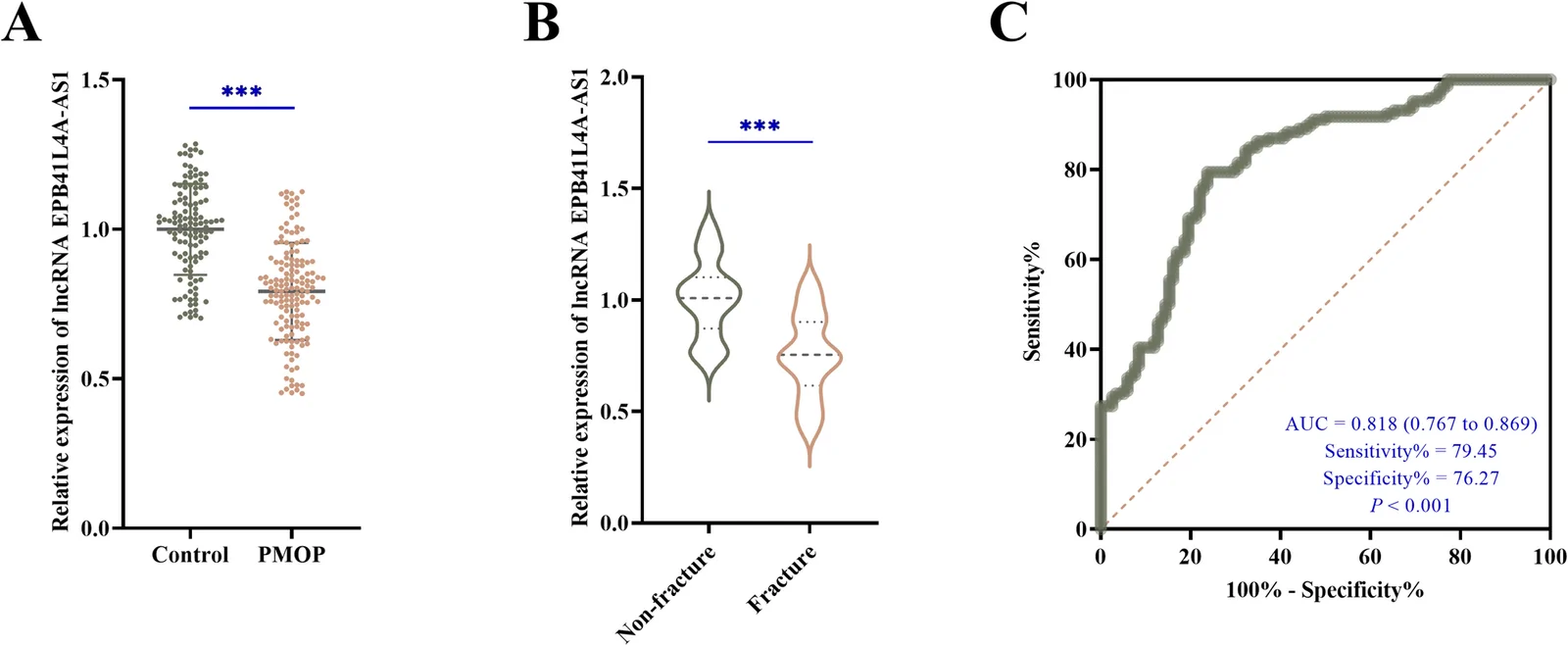
The serum EPB41L4A-AS1 expression in PMOP (**A**) and PMOF patients (**B**). The ROC curve of serum EPB41L4A-AS1 for PMOP (**C**) diagnosis. **A** Comparison between control and PMOP groups was performed using an independent t‑test; P values are shown. **B** Comparisons among non‑fracture and fracture subgroups were performed using independent t‑test; *P* values are shown. **C** ROC curve analysis with 95% CI; AUC comparison was performed using the DeLong test. Data are presented as mean ± standard deviation (SD). ***, *P* < 0.001

Serum levels of EPB41L4A-AS1 were markedly decreased in PMOP patients (*P* < 0.001, Fig. 1A), and further decreased in the fracture subgroup (*P* < 0.001, Fig. 1B).

The AUC of serum EPB41L4A-AS1 for distinguishing healthy individuals from PMOP patients was 0.818, with a sensitivity of 79.45% and a specificity of 76.27% (*P* < 0.001, Fig. 1C). To further evaluate the incremental diagnostic value of EPB41L4A-AS1, this study compared its performance with established bone metabolism markers and clinical parameters. As shown in Supplementary Table 2, the AUCs of conventional markers were notably lower: β-CTX (AUC = 0.677, sensitivity = 65.07%, specificity = 70.34%), OCN (AUC = 0.640, sensitivity = 60.27%, specificity = 67.80%), and T scores (AUC = 0.733, sensitivity = 71.23%, specificity = 75.42%). EPB41L4A-AS1 significantly outperformed β-CTX, OCN, and T scores in discriminating PMOP patients from healthy controls.

Furthermore, this study constructed a combined diagnostic model incorporating EPB41L4A-AS1 together with β-CTX, OCN, and T scores. The combined model demonstrated a substantially improved diagnostic performance with an AUC of 0.913, sensitivity of 74.66%, and specificity of 89.83% (*P* < 0.001, Supplementary Table 2).

### The correlation between serum EPB41L4A-AS1 and clinical indicators

In PMOP patients, serum EPB41L4A-AS1 expression was significantly positively correlated with L1-4 BMD (*r* = 0.646), FN BMD (*r* = 0.605), TH BMD (*r* = 0.671), and T value (*r* = 0.683) (*P* < 0.001). For serum biochemical indicators, EPB41L4A-AS1 was positively correlated with 25(OH)D₃ (*r* = 0.446), Ca²⁺ (*r* = 0.415), and E2 (*r* = 0.494), and negatively correlated with β-CTX (*r* = -0.568) and OCN (*r* = -0.549) (*P* < 0.001). These results indicated that serum EPB41L4A-AS1 expression was closely associated with bone metabolic balance (Table 2).

**Table 2:** Correlation values between serum EPB41L4A-AS1 and clinical indicators in PMOP patients

Parameters	*r* (95% CI)	*P* value
L1-4 BMD, g/cm^2^	0.646 (0.540 to 0.732)	< 0.001
FN BMD, g/cm^2^	0.605 (0.491 to 0.699)	< 0.001
TH BMD, g/cm^2^	0.671 (0.571 to 0.752)	< 0.001
T scores	0.683 (0.586 to 0.761)	< 0.001
25(OH)D_3_, ng/mL	0.446 (0.305 to 0.567)	< 0.001
Ca^2+^, mmol/L	0.415 (0.271 to 0.541)	< 0.001
β-CTX, ng/mL	-0.568 (-0.669 to -0.447)	< 0.001
OCN, ng/mL	-0.549 (-0.653 to -0.424)	< 0.001
E2, pmol/L	0.494 (0.361 to 0.608)	< 0.001

*Abbreviations* *PMOP* postmenopausal osteoporosis, *95% CI* 95% confidence interval, *L1-4 BMD* lumbar spine bone mineral density, *FN BMD * femoral neck bone mineral density, *TH BMD * total hip bone mineral density, *25(OH)*D_3_ 25-hydroxyvitamin D_3_, *Ca*^2+^ calcium ion, *β-CTX * β-isomerized C-terminal telopeptide of type I collagen, *OCN * osteocalcin, *E2 * Estradiol

### The predictive significance of serum EPB41L4A-AS1 for PMOP and fracture occurrence

**Fig. 2:**
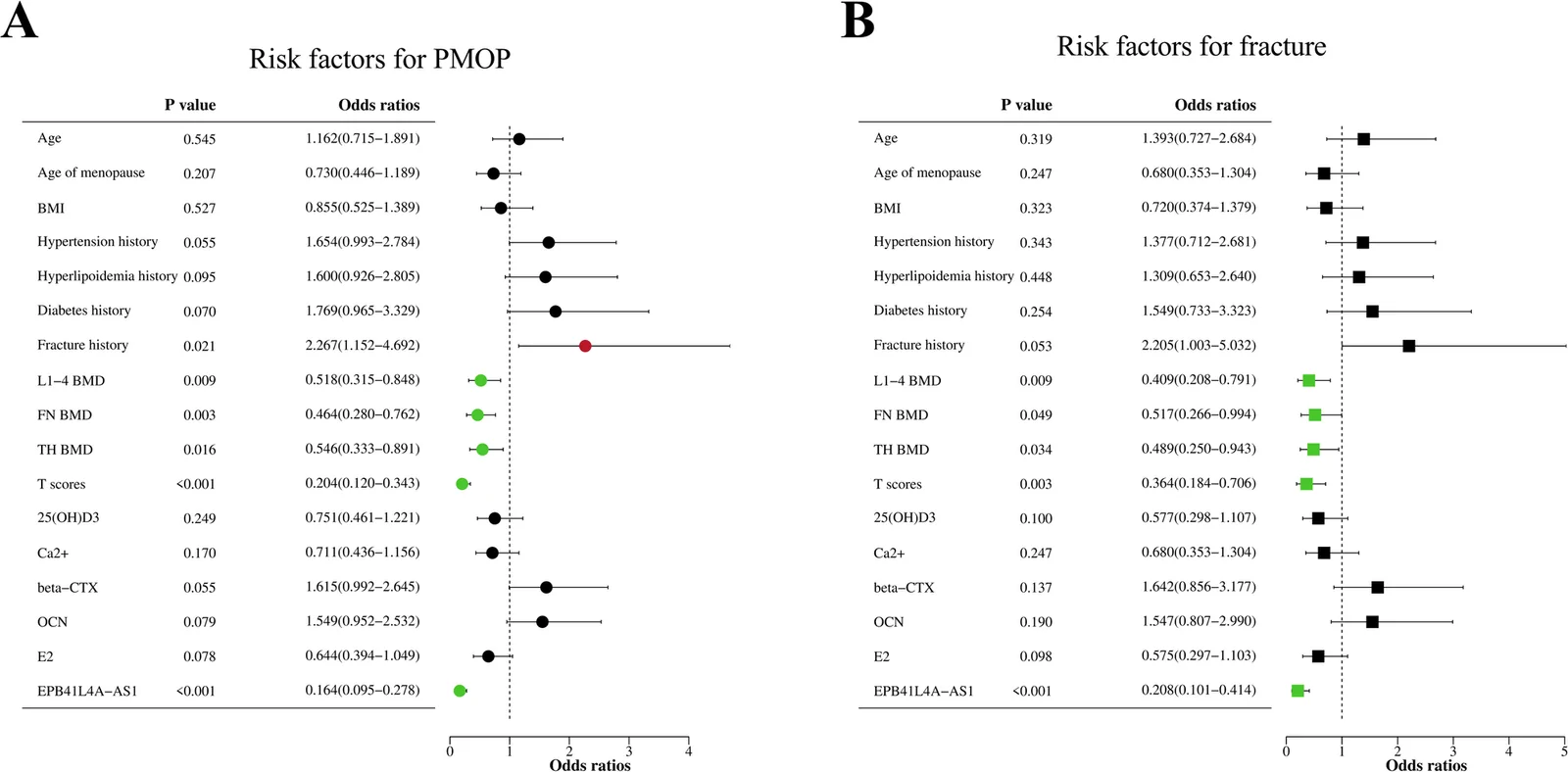
Prediction of risk factors associated with the occurrence of PMOP (**A**) and PMOF (**B**) via binary logistic regression models. ORs and 95% CIs are presented

To assess the independent association of EPB41L4A-AS1 with PMOP and fragility fractures, this study performed multivariate binary logistic regression adjusting for established clinical and bone densitometric confounders. Covariates included age, age at menopause, BMI, hypertension, hyperlipidemia, diabetes, lumbar spine BMD, femoral neck BMD, total hip BMD, T-score, and clinical blood indicators—selected a priori based on clinical relevance and retained in the final model following rigorous variable screening. Logistic regression analysis identified low serum levels of EPB41L4A-AS1 (OR = 0.164, *P* < 0.001) and reduced T-scores (OR = 0.204, *P* < 0.001) as the most strongly associated independent predictors of PMOP occurrence. Furthermore, decreased BMD values at the L1–L4 (*P* = 0.009), FN (*P* = 0.003), and TH (*P* = 0.016), along with a history of fractures, were significantly linked to higher PMOP risk (Fig. 2A).

Subsequent regression modeling assessing fracture risk among PMOP patients indicated that downregulated serum EPB41L4A-AS1 was the most prominen risk factor for fracture (*P* < 0.001). Decreased T scores (*P* = 0.003) and BMD levels (*P* = 0.009; *P* = 0.049; *P* = 0.034) were also significantly correlated with increased fracture risk (Fig. 2B). Collectively, low EPB41L4A-AS1 expression exhibited the strongest predictive ability for both PMOP onset and fracture occurrence.

#### The effect of silencing EPB41L4A-AS1 on osteogenic differentiation of ADSCs and bone microenvironment

**Fig. 3:**
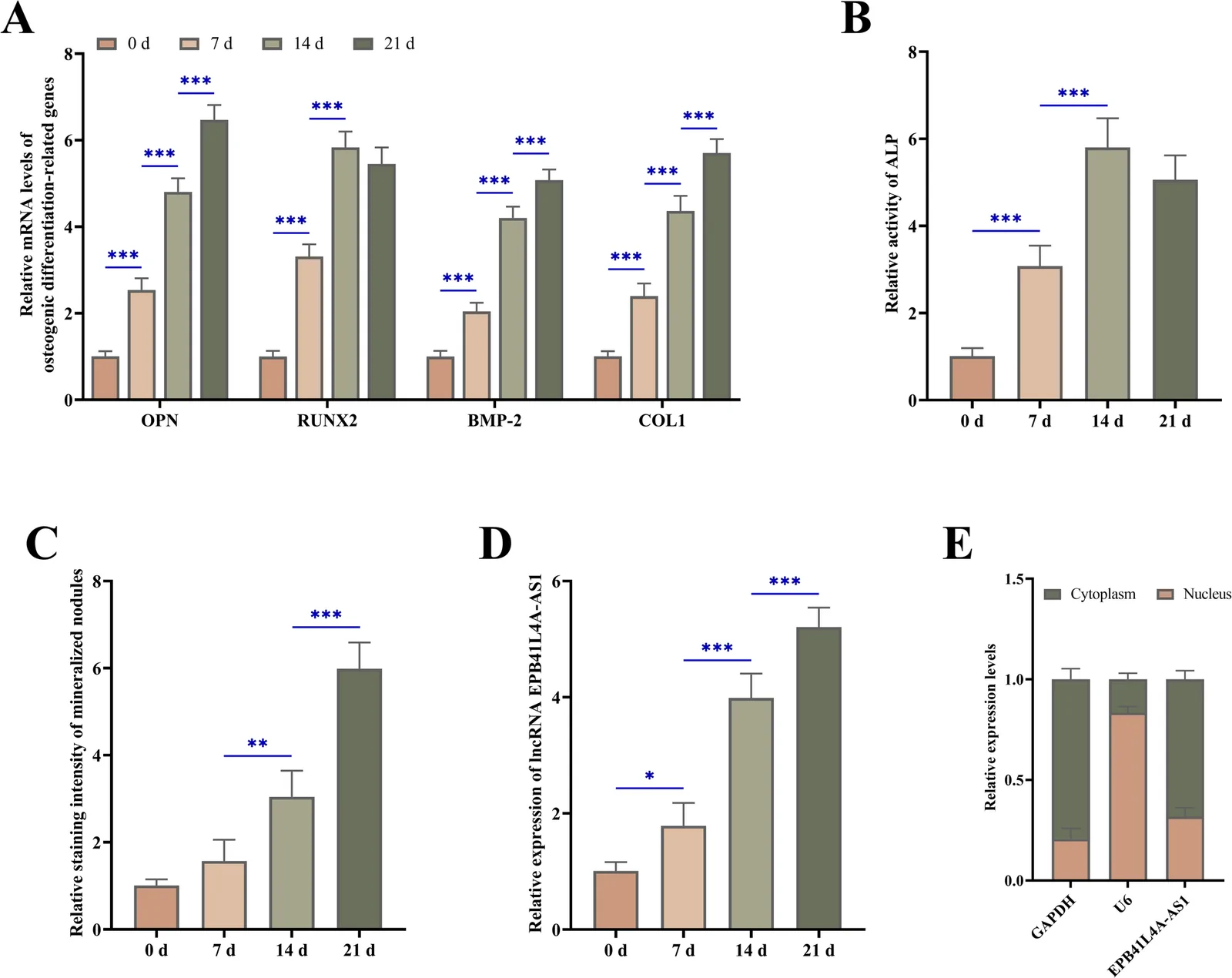
The mRNA levels of OPN, RUNX2, BMP-2, and COL1 (**A**), the activity of ALP (**B**), the staining intensity of mineralized nodules (**C**), as well as the level of EPB41L4A-AS1 expression (**D**) during the induction of ADSC osteogenic differentiation. The relative expression levels of EPB41L4A-AS1 in the cytoplasm and nucleus of ADSCs (**E**). Data were analyzed using repeated‑measures ANOVA with Bonferroni post‑hoc pairwise comparisons. *P* values for each comparison are provided in the figure. Data are presented as mean ± SD. *, *P* < 0.05; **, *P* < 0.01; ***, *P* < 0.001

After osteogenic induction, the mRNA expression of the osteogenic differentiation-related genes OPN, RUNX2, BMP-2, and COL1 increased gradually with induction time (*P* < 0.001, Fig. 3A). ALP activity continued to rise during induction and was significantly higher on day 21 than at baseline (*P* < 0.001, Fig. 3B). ARS staining showed that the number of mineralized nodules increased in a time-dependent manner, reaching the peak on day 21 (*P* < 0.01, Fig. 3C). At the same time, EPB41L4A-AS1 expression was gradually increased during ADSC osteogenic differentiation (*P* < 0.05, Fig. 3D). Nuclear-cytoplasmic fractionation experiments revealed that EPB41L4A-AS1 was mainly distributed in the cytoplasm of ADSCs (Fig. 3E).

**Fig. 4:**
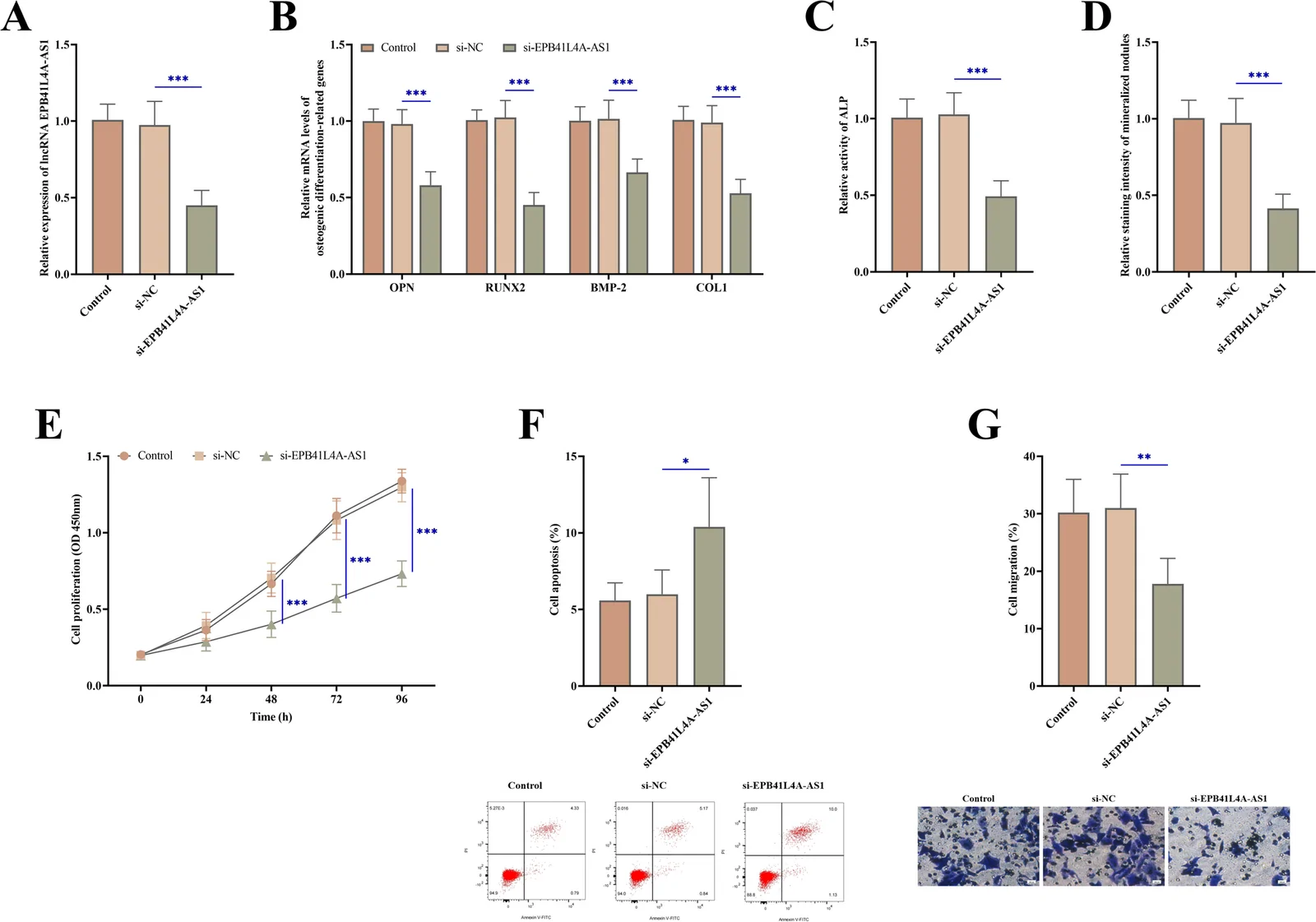
The effects of silencing EPB41L4A-AS1 in osteogenic differentiation cell model on EPB41L4A-AS1 expression (**A**), the mRNA levels of osteogenic differentiation-related genes (**B**), the ALP activity (**C**), the staining intensity of mineralized nodules (**D**), as well as the proliferative activity (**E**), apoptosis rate (**F**), and migration rate (**G**) of ADSCs. Data were analyzed using repeated‑measures ANOVA with Bonferroni post‑hoc pairwise comparisons. *P* values for each comparison are provided in the figure. Data are presented as mean ± SD. *, *P* < 0.05; **, *P* < 0.01; ***, *P* < 0.001

After silencing EPB41L4A-AS1 in the osteogenic differentiation model, the expression of EPB41L4A-AS1 was significantly decreased (*P* < 0.001, Fig. 4A). The mRNA expression levels of OPN, RUNX2, BMP-2, and COL1 were significantly downregulated (*P* < 0.001, Fig. 4B), accompanied by inhibited ALP activity (*P* < 0.001, Fig. 4C) and reduced mineralized nodule formation (*P* < 0.001, Fig. 4D).

Cell function experiments revealed that silencing EPB41L4A-AS1 significantly inhibited ADSC proliferation at 48, 72 and 96 h (*P* < 0.001, Fig. 4E), increased cell apoptosis rate (*P* < 0.05, Fig. 4F) and impaired cell migration ability (*P* < 0.01, Fig. 4G).

**Fig. 5:**
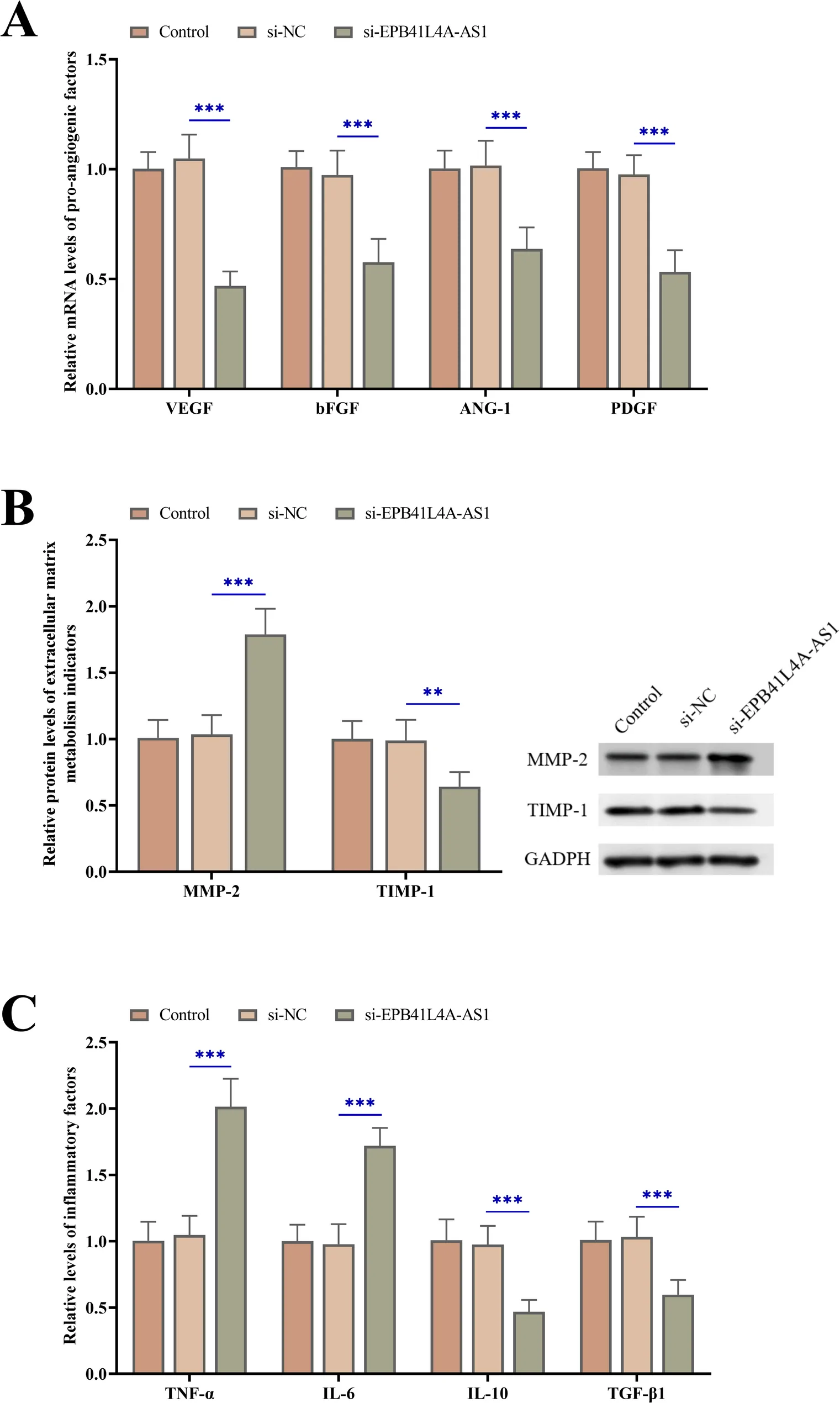
The effects of silencing EPB41L4A-AS1 in osteogenic differentiation cell model on the mRNA levels of pro-angiogenic factors VEGF, bFGF, ANG-1, and PDGF (**A**), the protein levels of extracellular matrix remodeling markers MMP-2 and TIMP-1 (**B**), and the protein levels of inflammatory factors TNF-α, IL-6, IL-10, and TGF-β1 (**C**). Data were analyzed using repeated‑measures ANOVA with Bonferroni post‑hoc pairwise comparisons. *P *values for each comparison are provided in the figure. Data are presented as mean ± SD. **, *P* < 0.01; ***, *P* < 0.001

Upon silencing of EPB41L4A-AS1, a significant downregulation was observed in the mRNA expression of key angiogenic factors—VEGF, bFGF, ANG-1, and PDGF—in ADSCs (*P* < 0.001, Fig. 5A). Analysis of extracellular matrix remodeling markers revealed a marked increase in MMP-2 protein levels, accompanied by a significant reduction in TIMP-1 expression (*P* < 0.01, Fig. 5B, Supplementary attachment 1). With respect to inflammatory mediators, the protein secretion of pro-inflammatory cytokines TNF-α and IL-6 was significantly elevated, whereas levels of the anti-inflammatory cytokines IL-10 and TGF-β1 were substantially reduced (*P* < 0.001, Fig. 5C).

### Verification of the targeting relationship between EPB41L4A-AS1 and miR-302d-3p

**Fig. 6:**
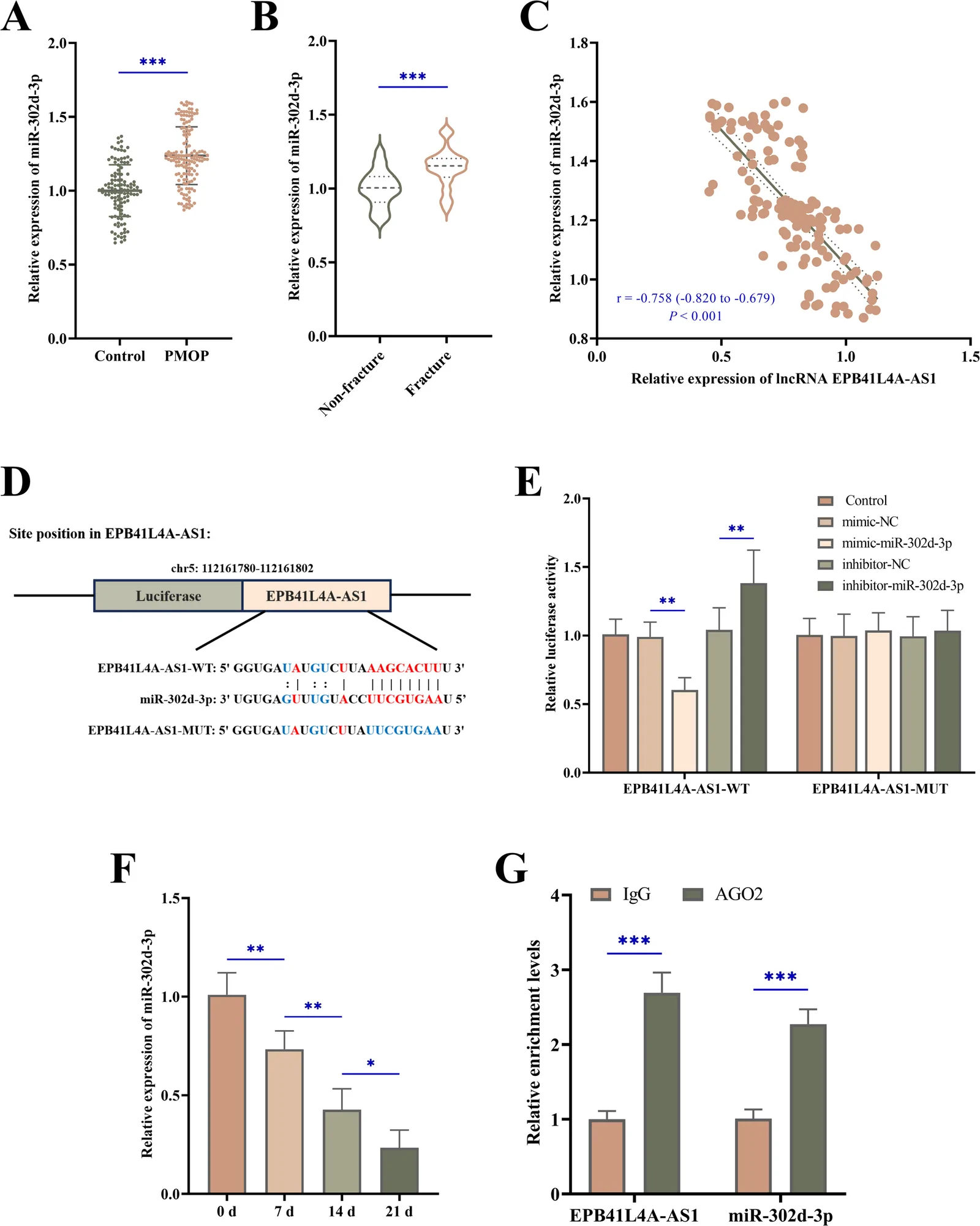
The serum miR-302d-3p expression in PMOP (**A**) and PMOF patients (**B**), and the correlation between EPB41L4A-AS1 and miR-302d-3p expression in PMOP patients (**C**). Prediction results of the binding sites of EPB41L4A-AS1 and miR-302d-3p (**D**) and verification of the targeted binding relationship between EPB41L4A-AS1 and miR-302d-3p (**E**). The level of miR-302d-3p expression (**F**) during the induction of ADSC osteogenic differentiation. RIP detected the EPB41L4A-AS1 and miR-302d-3p expressions pulled down by AGO2 in ADSCs (**G**). **A** Comparison between control and PMOP groups was performed using an independent t‑test; *P* values are shown. **B** Comparisons among non‑fracture and fracture subgroups were performed using independent t‑test; *P* values are shown. **C** Pearson rank correlation with exact r and *P* values. **E** Luciferase activity was analyzed using two‑way ANOVA with Bonferroni post‑hoc pairwise comparisons for pairwise contrasts between WT and MUT vectors. **F** Repeated‑measures ANOVA with Bonferroni post‑hoc test. P values are indicated. Data are presented as mean ± SD. *, *P* < 0.05; **, *P* < 0.01; ***, *P* < 0.001

Serum detection results revealed that PMOP patients exhibited significantly higher levels of miR-302d-3p expression (*P* < 0.001, Fig. 6A), and was further upregulated in the fracture subgroup (*P* < 0.001, Fig. 6B). A significant negative correlation was observed between the expression of miR-302d-3p and EPB41L4A-AS1 in the serum of PMOP patients (*r* = -0.758, *P* < 0.001, Fig. 6C).

Bioinformatic analysis using StarBase predicted a potential complementary binding site between EPB41L4A-AS1 and miR-302d-3p (Fig. 6D). Dual-luciferase reporter assays confirmed this interaction. The miR-302d-3p mimic significantly suppressed the luciferase activity of the EPB41L4A-AS1 wild-type vector, whereas the miR-302d-3p inhibitor markedly enhanced it (*P* < 0.01). In contrast, no significant change in luciferase activity was observed in the mutant vector group (Fig. 6E).

During osteogenic induction, miR-302d-3p expression in ADSCs progressively decreased over time (*P* < 0.01), exhibiting an inverse expression pattern relative to EPB41L4A-AS1 (Fig. 6F). Furthermore, the AGO2-RIP experimental results in ADSCs demonstrated that, compared with the IgG control group, EPB41L4A-AS1 was significantly enriched in the RNA-induced silencing complex (RISC) complex following antibody precipitation, and miR-302d-3p was specifically co-precipitated (*P* < 0.001, Fig. 6G).

#### EPB41L4A-AS1 regulates osteogenic differentiation of ADSCs and bone microenvironment via miR-302d-3p

**Fig. 7:**
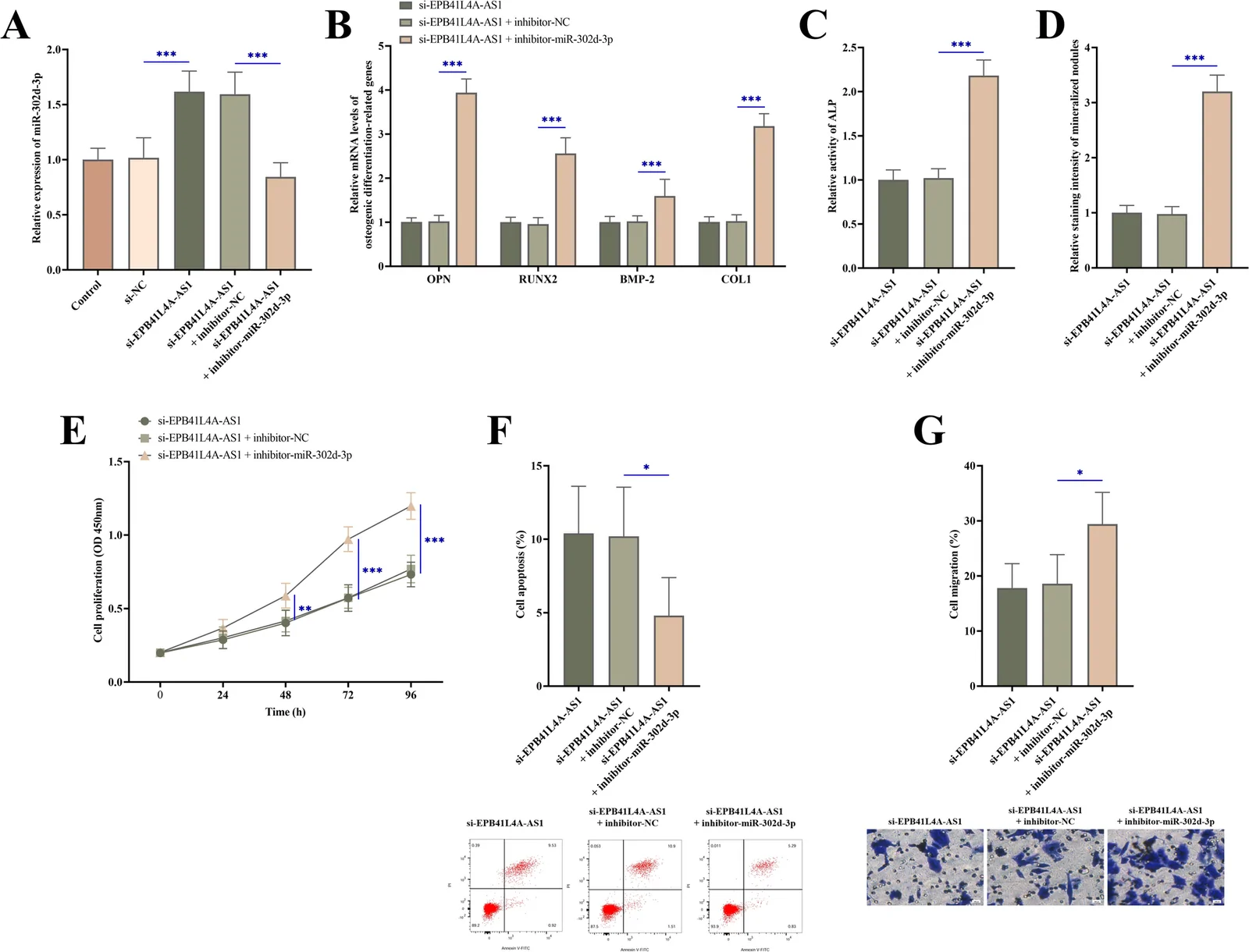
The effects of EPB41L4A-AS1 and miR-302d-3p co-inhibition in osteogenic differentiation cell model on miR-302d-3p expression (**A**), the mRNA levels of osteogenic differentiation-related genes (**B**), the ALP activity (**C**), the staining intensity of mineralized nodules (**D**), as well as the proliferative activity (**E**), apoptosis rate (**F**), and migration rate (**G**) of ADSCs. Data were analyzed using repeated‑measures ANOVA with Bonferroni post‑hoc pairwise comparisons. *P* values for each comparison are provided in the figure. Data are presented as mean ± SD. *, *P* < 0.05; **, *P* < 0.01; ***, *P* < 0.001

Knockdown of EPB41L4A-AS1 alone in the osteogenic differentiation model, the expression level of miR-302d-3p in ADSCs increased significantly (*P* < 0.001). Conversely, simultaneous inhibition of EPB41L4A-AS1 and miR-302d-3p significantly reduced the expression of the latter (*P* < 0.001, Fig. 7A). Co-silencing EPB41L4A-AS1 and miR-302d-3p effectively restored the osteogenic differentiation capacity of ADSCs, as evidenced by significant upregulation of the expression levels of osteogenic differentiation-related genes (*P* < 0.001, Fig. 7B), a significant enhance in ALP activity (*P* < 0.001, Fig. 7C) and a significant increase in the degree of mineralization (*P* < 0.001, Fig. 7D).

The ADSC dysfunction caused by EPB41L4A-AS1 silencing was substantially alleviated upon dual inhibition of EPB41L4A-AS1 and miR-302d-3p. Cell proliferation activity in the co-silencing group was significantly restored (*P* < 0.01, Fig. 7E), the apoptosis rates were distinctly reduced (*P* < 0.05, Fig. 7F) and cell migration ability was significantly enhanced (*P* < 0.05, Fig. 7G).

**Fig. 8:**
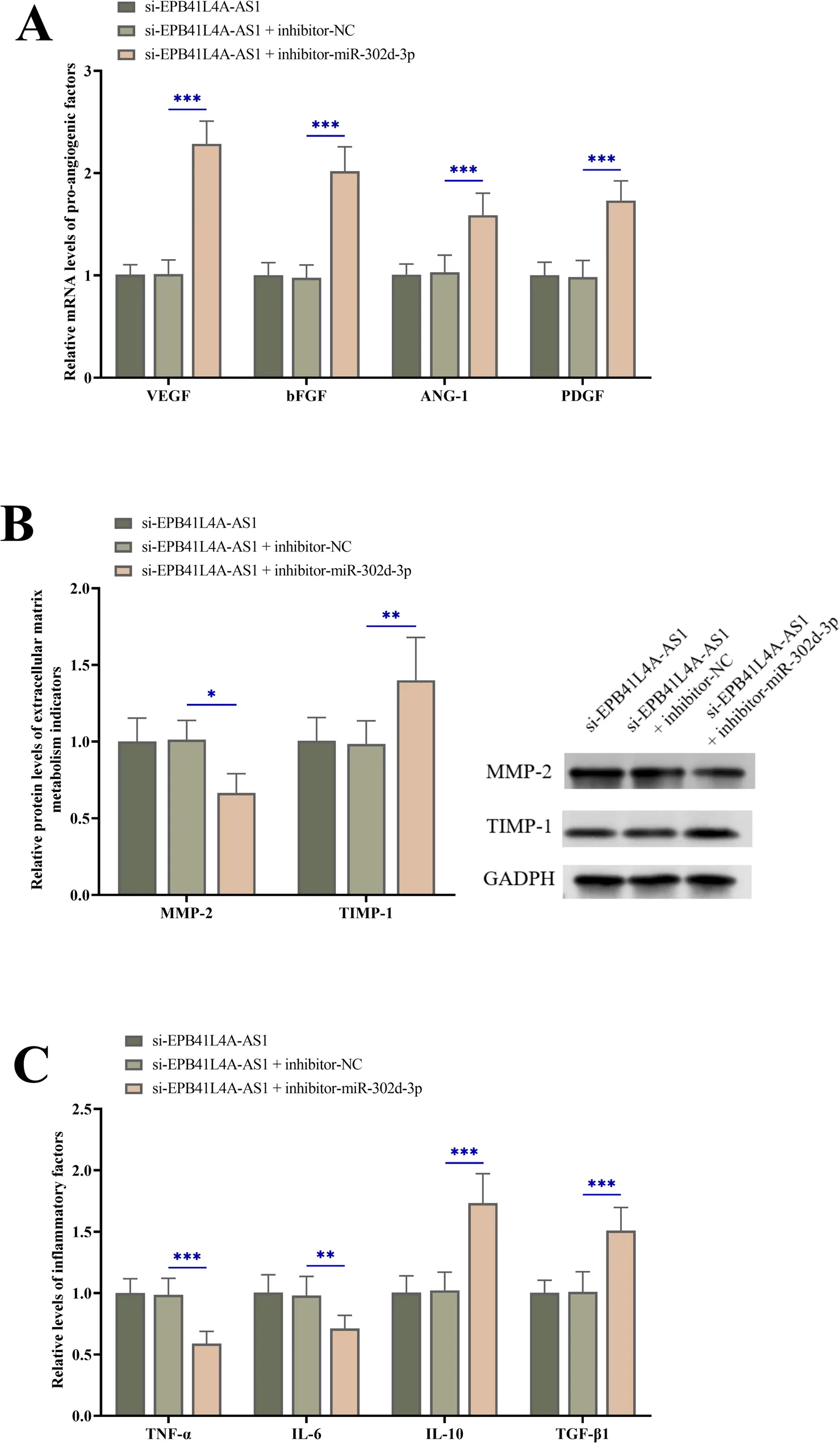
The effects of EPB41L4A-AS1 and miR-302d-3p co-inhibition in osteogenic differentiation cell model on the mRNA levels of pro-angiogenic factors VEGF, bFGF, ANG-1, and PDGF (**A**), the protein levels of extracellular matrix remodeling markers MMP-2 and TIMP-1 (**B**), and the protein levels of inflammatory factors TNF-α, IL-6, IL-10, and TGF-β1 (**C**). Data were analyzed using repeated‑measures ANOVA with Bonferroni post‑hoc pairwise comparisons. *P* values for each comparison are provided in the figure. Data are presented as mean ± SD. **, *P* < 0.01; ***, *P* < 0.001

Regarding modulation of the bone microenvironment, combined suppression of EPB41L4A-AS1 and miR-302d-3p significantly increase the levels of angiogenic factors (*P* < 0.001, Fig. 8A). Meanwhile, the protein levels of extracellular matrix remodeling markers also altered. MMP-2 expression was markedly reduced, while TIMP-1 showed a significant upregulation (*P* < 0.05, Fig. 8B, Supplementary attachment 1). This dual inhibition also restored inflammatory balance by substantially decreasing the secretion of pro-inflammatory cytokines from ADSCs, while enhancing the production of anti-inflammatory factors (*P* < 0.01, Fig. 8C). Although silencing EPB41L4A-AS1 alone impairs osteogenic differentiation of ADSCs and destabilizes the bone microenvironment, these adverse effects were effectively counteracted by concurrent inhibition of miR-302d-3p.

#### Functional validation of miR-302d-3p as a downstream mediator of EPB41L4A-AS1

To functionally validate whether miR-302d-3p mediates the biological effects of EPB41L4A-AS1, ADSCs undergoing osteogenic induction were transfected with miR-302d-3p mimic or mimic-NC. qPCR confirmed that miR-302d-3p expression was markedly elevated in the mimic group compared with the control and mimic-NC groups (*P* < 0.001, Supplementary Fig. 1A). Overexpression of miR-302d-3p significantly suppressed the mRNA expression of osteogenic differentiation-related genes OPN, RUNX2, BMP-2, and COL1 (*P* < 0.01, Supplementary Fig. 1B), reduced ALP activity (*P* < 0.01, Supplementary Fig. 1C), and decreased mineralized nodule formation (*P* < 0.001, Supplementary Fig. 1D). Functionally, miR-302d-3p mimic significantly inhibited ADSC proliferation at 48, 72, and 96 h (*P* < 0.01, Supplementary Fig. 1E), increased the apoptosis rate (*P* < 0.05, Supplementary Fig. 1F), and impaired cell migration (*P* < 0.01, Supplementary Fig. 1G).

Regarding modulation of the bone microenvironment, miR-302d-3p overexpression significantly downregulated the expression of pro-angiogenic factors VEGF, bFGF, ANG-1, and PDGF (*P* < 0.01, Supplementary Fig. 2A). Extracellular matrix remodeling was disrupted, as evidenced by elevated MMP-2 protein levels and decreased TIMP-1 expression (*P* < 0.01, Supplementary Fig. 2B). Furthermore, the inflammatory balance shifted toward a pro-inflammatory phenotype, with increased secretion of TNF-α and IL-6 and reduced levels of IL-10 and TGF-β1 (*P* < 0.01, Supplementary Fig. 2C). Collectively, these phenotypic changes closely mirrored those induced by EPB41L4A-AS1 silencing, functionally validating miR-302d-3p as a key downstream mediator of EPB41L4A-AS1 in regulating ADSC osteogenic differentiation and bone microenvironment homeostasis.

#### Identification of CUL3 as a direct downstream target of miR-302d-3p

**Fig. 9:**
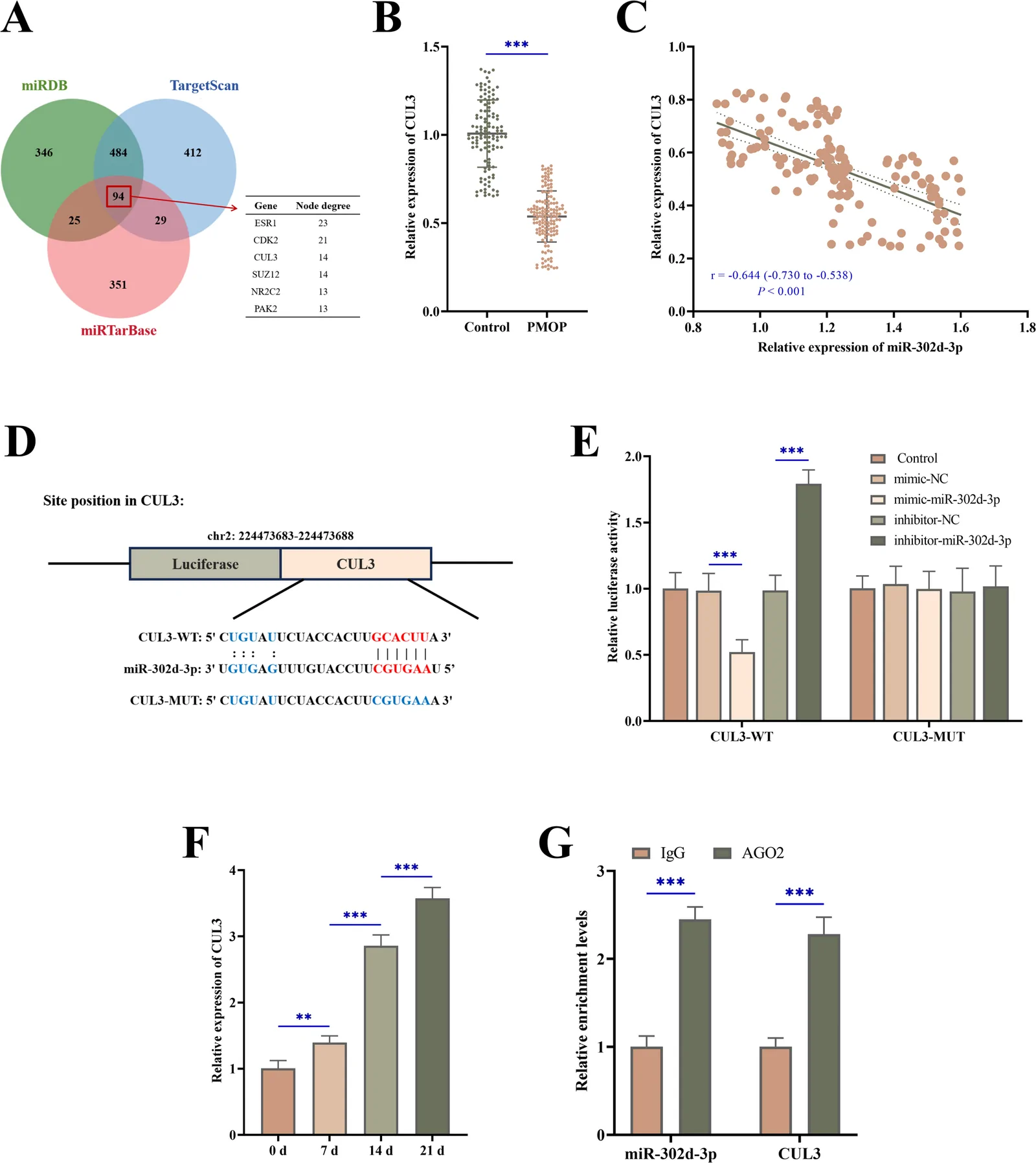
Venn diagram showing the intersection of miR-302d-3p target genes predicted by miRDB, TargetScan, and miRTarBase; CUL3 exhibited higher node degree (**A**). Serum CUL3 mRNA levels were significantly downregulated in PMOP patients compared with controls (**B**). Pearson correlation analysis revealed a significant negative correlation between serum miR-302d-3p and CUL3 levels in PMOP patients (**C**). Schematic illustration of the predicted binding site between miR-302d-3p and the 3′-UTR of CUL3, along with the mutated sequence (**D**). Dual-luciferase reporter assay confirming the direct targeting relationship (**E**). CUL3 expression progressively increased during osteogenic induction of ADSCs (**F**). RIP detected the miR-302d-3p and CUL3 expressions pulled down by AGO2 in ADSCs (**G**). **B** Comparison between control and PMOP groups was performed using an independent t‑test; P values are shown. **C** Pearson rank correlation with exact r and *P* values. **E** Luciferase activity was analyzed using two‑way ANOVA with Bonferroni post‑hoc pairwise comparisons for pairwise contrasts between WT and MUT vectors. **F** Repeated‑measures ANOVA with Bonferroni post‑hoc test. P values are indicated. Data are presented as mean ± SD. **, *P* < 0.01; ***, *P* < 0.001

To elucidate the downstream mechanism of the EPB41L4A-AS1/miR-302d-3p axis, bioinformatics analysis was performed to predict miR-302d-3p target genes. Intersection of the miRDB, TargetScan, and miRTarBase databases identified CUL3 as a high-confidence candidate with higher node degree (Fig. 9A). Serum qPCR analysis revealed that CUL3 mRNA levels were significantly downregulated in PMOP patients compared with healthy controls (*P* < 0.001, Fig. 9B). Moreover, a significant negative correlation was observed between serum miR-302d-3p and CUL3 expression in PMOP patients (*r* = -0.644, *P* < 0.001, Fig. 9C).

The predicted complementary binding site between miR-302d-3p and the 3′-UTR of CUL3 is illustrated in Fig. 9D. Dual-luciferase reporter assays confirmed this direct targeting interaction: the miR-302d-3p mimic significantly suppressed the luciferase activity of the WT CUL3 vector, whereas the miR-302d-3p inhibitor markedly enhanced it (*P* < 0.001); no significant change was observed with the MUT construct (Fig. 9E). During osteogenic induction of ADSCs, CUL3 expression progressively increased over time (*P* < 0.01, Fig. 9F), paralleling the upregulation of EPB41L4A-AS1 and inversely mirroring the dynamics of miR-302d-3p. Additionally, the AGO2-RIP experiments conducted in ADSCs showed that, compared to the IgG control group, miR-302d-3p was significantly enriched in the RISC after antibody precipitation, and CUL3 was specifically co-precipitated (*P* < 0.001, Fig. 9G).

### CUL3 functions as the downstream effector of the EPB41L4A-AS1/miR-302d-3p axis

**Fig. 10:**
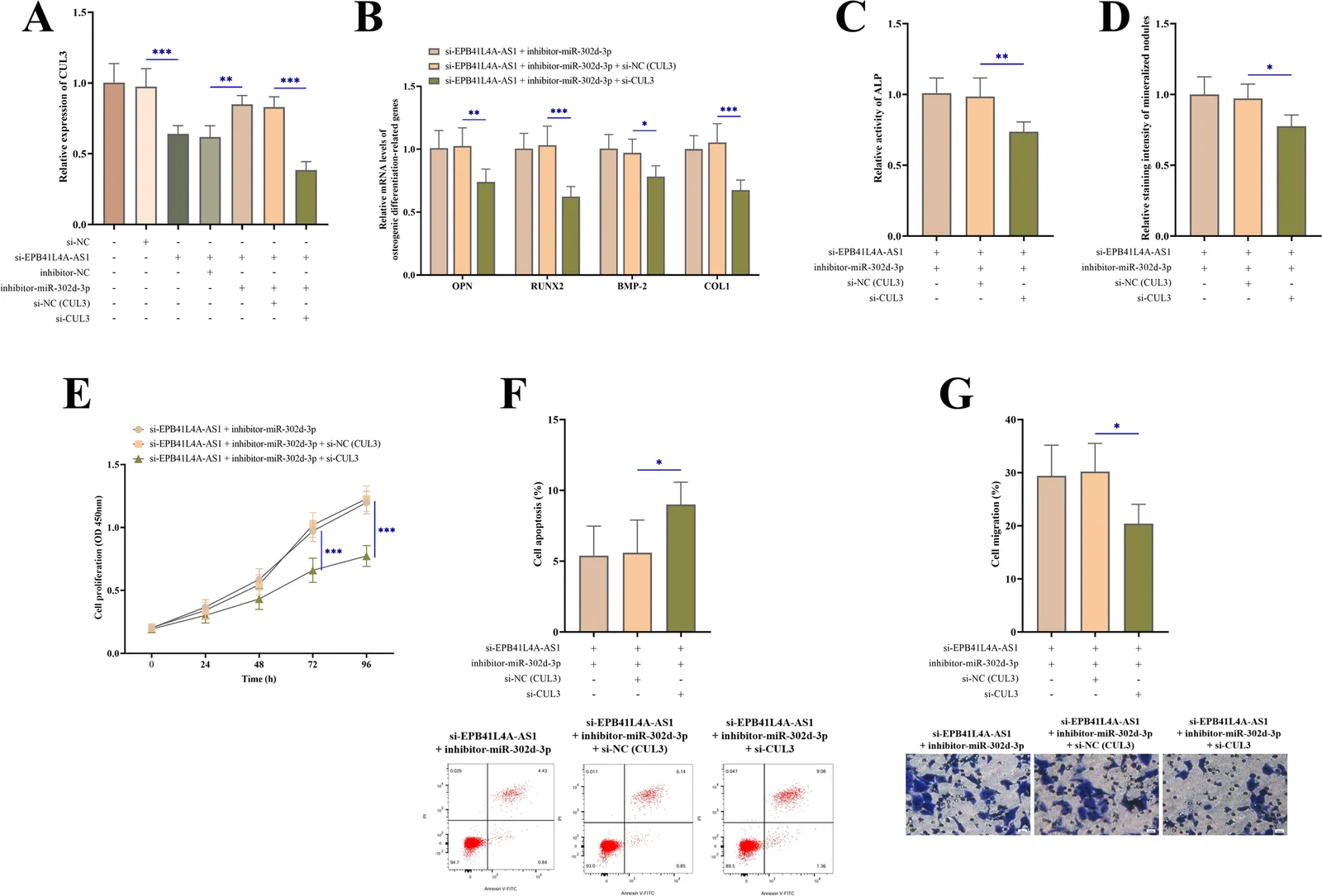
The effects of EPB41L4A-AS1, miR-302d-3p, and CUL3 co-inhibition in osteogenic differentiation cell model on CUL3 mRNA expression (**A**), the mRNA levels of osteogenic differentiation-related genes (**B**), the ALP activity (**C**), the staining intensity of mineralized nodules (**D**), as well as the proliferative activity (**E**), apoptosis rate (**F**), and migration rate (**G**) of ADSCs. Data were analyzed using repeated‑measures ANOVA with Bonferroni post‑hoc pairwise comparisons. *P* values for each comparison are provided in the figure. Data are presented as mean ± SD. *, *P* < 0.05; **, *P* < 0.01; ***, *P* < 0.001

To determine whether CUL3 mediates the biological effects of the EPB41L4A-AS1/miR-302d-3p axis, rescue-reversion experiments were performed. In ADSCs co-transfected with si-EPB41L4A-AS1 and inhibitor-miR-302d-3p, CUL3 expression was maintained at levels comparable to the control group; however, additional knockdown of CUL3 (si-CUL3) significantly reduced its expression (*P* < 0.001, Fig. 10A).

Functionally, while co-inhibition of EPB41L4A-AS1 and miR-302d-3p rescued osteogenic differentiation, subsequent CUL3 silencing reversed these beneficial effects. The mRNA levels of OPN, RUNX2, BMP-2, and COL1 were significantly downregulated (*P* < 0.05, Fig. 10B), ALP activity was markedly reduced (*P* < 0.01, Fig. 10C), and mineralized nodule formation was decreased (*P* < 0.05, Fig. 10D) in the CUL3 silencing group.

Cellular function assays showed that CUL3 knockdown significantly inhibited ADSC proliferation at 72 and 96 h (*P* < 0.001, Fig. 10E), increased the apoptosis rate (*P* < 0.05, Fig. 10F), and impaired cell migration (*P* < 0.05, Fig. 10G).

**Fig. 11:**
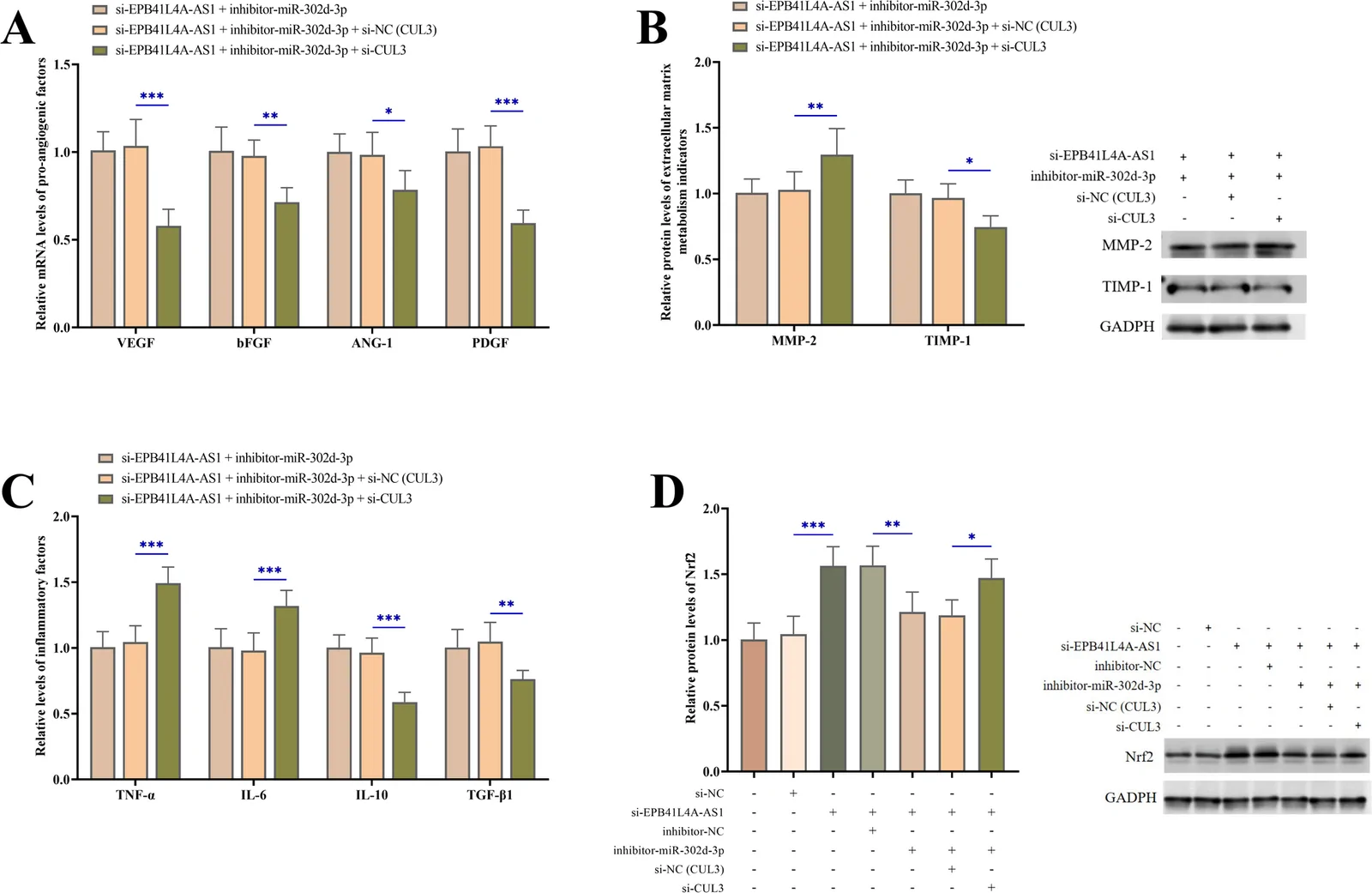
The effects of EPB41L4A-AS1, miR-302d-3p, and CUL3 co-inhibition in osteogenic differentiation cell model on the mRNA levels of pro-angiogenic factors VEGF, bFGF, ANG-1, and PDGF (**A**), the protein levels of extracellular matrix remodeling markers MMP-2 and TIMP-1 (**B**), and the protein levels of inflammatory factors TNF-α, IL-6, IL-10, and TGF-β1 (**C**), and the protein levels of Nrf2 (**D**). Data were analyzed using repeated‑measures ANOVA with Bonferroni post‑hoc pairwise comparisons. *P* values for each comparison are provided in the figure. Data are presented as mean ± SD. *, *P* < 0.05; **, *P* < 0.01; ***, *P* < 0.001

Regarding the bone microenvironment, CUL3 silencing significantly downregulated the expression of angiogenic factors including VEGF, bFGF, ANG-1, and PDGF (*P* < 0.05, Fig. 11A). Extracellular matrix remodeling was also disrupted, as evidenced by elevated MMP-2 protein levels and decreased TIMP-1 expression (*P* < 0.05, Fig. 11B, Supplementary attachment 1). Furthermore, the inflammatory balance was shifted toward a pro-inflammatory phenotype, with increased secretion of TNF-α and IL-6 and reduced levels of IL-10 and TGF-β1 (*P* < 0.01, Fig. 11C).

To elucidate the molecular bridge linking CUL3 to the observed bone microenvironmental phenotypes, we assessed Nrf2 protein levels across the rescue-reversion experimental groups. Western blot analysis revealed that EPB41L4A-AS1 silencing alone significantly elevated Nrf2 protein abundance compared with the control group (*P* < 0.001). Conversely, co-inhibition of miR-302d-3p in the EPB41L4A-AS1-silenced background restored Nrf2 protein levels to near-baseline values (*P* < 0.01). Importantly, additional CUL3 knockdown under this rescued condition significantly re-elevated Nrf2 protein expression (*P* < 0.05), establishing an inverse correlation between CUL3 and Nrf2 protein stability downstream of the EPB41L4A-AS1/miR-302d-3p axis (Fig. 11D, Supplementary attachment 1).

## Discussion

PMOP is a complex degenerative bone disease. The secondary osteoporotic fractures can cause irreversible motor dysfunction and bring a heavy public health burden [20]. Current clinical approaches fail to effectively identify early molecular warnings and correct underlying bone metabolism disorders [21]. Therefore, exploring key regulatory molecules is crucial to break through the limitations of existing diagnostic and treatment strategies, and realize targeted interventions.

LncRNAs have the advantages of high sensitivity, non-invasive detection and multidimensional gene regulation, providing new directions for disease mechanism research and clinical transformation in PMOP [22]. Previous studies have confirmed that lncRNAs such as MALAT1, MIR155HG, and lncTIMP3 can regulate stem cell osteogenic differentiation or bone metabolic balance, and are expected to become diagnostic and therapeutic targets for PMOP [23–25]. EPB41L4A-AS1 is a potential biomarker for ageing and age-related diseases [10] and participates in the regulation of various cellular biological processes [5–7]. This study found that serum EPB41L4A-AS1 expression was low in PMOP patients and further reduced in the fracture subgroup. The molecule showed high diagnostic sensitivity and specificity in differentiating PMOP patients from healthy individuals. Serum EPB41L4A-AS1 significantly outperformed β-CTX, OCN, and T scores in discriminating PMOP patients from healthy controls, and showed even better performance when combined with clinical indicators, indicating its potential as a serum biomarker for early auxiliary diagnosis and clinical stratification of PMOP. It is important to emphasize that the translational claims of this study are strictly limited to clinical biomarker discovery and in vitro mechanistic proof-of-concept; any inference regarding therapeutic targeting requires validation in appropriate in vivo models.

BMD and T-scores are core indicators for evaluating bone mass, and their reduction is closely linked to suppressed bone formation and increased bone resorption [26]. 25(OH)D₃ and serum calcium are important raw materials for bone mineralization precursor availability, and their reduction will impair matrix calcification and weaken bone strength [27]. Elevated β-CTX and OCN indicate excessive bone resorption, which accelerates bone loss and bone microarchitectural damage [28]. These abnormalities provide a critical basis for assessing bone metabolism and disease severity in PMOP patients. This study found that serum EPB41L4A-AS1 expression was strongly correlated with BMD, T scores and multiple bone metabolism markers, indicating that EPB41L4A-AS1 might be involved in regulating the balance of bone metabolism and its expression could indirectly reflect the bone health status. Identifying the key risk factors for disease occurrence and progression is essential for achieving precise disease prevention and management [29]. This study further confirmed that low EPB41L4A-AS1 expression was a core independent factor influencing the occurrence of PMOP and increased fracture risk in patients, showing the strongest association. This finding highlights the association of EPB41L4A-AS1 with disease progression and supports its potential as a molecular indicator for PMOP risk stratification and fracture occurrence. Nevertheless, these observations remain correlational and require prospective validation before clinical application. However, this is a single-center study with all participants recruited from a single institution. The single-center design may introduce selection bias, as the study population may not fully represent the broader demographic and clinical diversity of PMOP patients across different geographic regions, ethnic backgrounds, and healthcare settings. Therefore, the generalizability of our findings—particularly the diagnostic cutoff values and predictive models for EPB41L4A-AS1—requires cautious interpretation. External validation in independent, multicenter cohorts with larger and more diverse patient populations is essential to confirm the robustness of serum EPB41L4A-AS1 as a diagnostic and fracture risk prediction biomarker. Although we demonstrated that serum GAPDH Ct values did not differ significantly among the three cohorts (Supplementary Fig. 3), the use of endogenous housekeeping genes for circulating RNA normalization remains debated, as their stability can theoretically be influenced by preanalytical variables such as hemolysis and exosome release dynamics. The absence of exogenous spike-in controls (e.g., cel-miR-39) represents a methodological limitation; while spike-ins effectively monitor technical variations during RNA extraction and reverse transcription, they cannot correct for inherent biological differences in circulating RNA concentrations across samples. Therefore, future multicenter validation studies should incorporate both exogenous spike-in controls and systematically validated endogenous reference genes to further improve the robustness of circulating RNA quantification.

ADSCs are widely available, highly proliferative, and possess strong osteogenic differentiation potential, serving as ideal seed cells for bone tissue repair and regeneration. Abnormal osteogenic differentiation of ADSCs and disordered bone microenvironment is important pathological characteristics of PMOP [9]. Gene expression related to osteogenic differentiation directly influences cellular osteogenic potential. ALP activity is a key marker of early differentiation [30], and mineralized nodule formation reflects the maturation efficiency of this process [31]. Together, these components form a complete osteogenic functional cascade, and their downregulation impairs bone formation and promotes PMOP progression. In this study, the above indicators and EPB41L4A-AS1 expression in ADSCs increased gradually during ADSC osteogenic induction, confirming the successful establishment of the in vitro osteogenic model and the positive regulatory effect of EPB41L4A-AS1 on osteogenic differentiation. After silencing EPB41L4A-AS1, the expression of osteogenic-related genes, ALP activity, and the degree of mineralization were significantly decreased, which further clarifying its promoting effect on the osteogenic differentiation of ADSCs. The proliferation, apoptosis and migration functions of ADSCs directly affect their colonization and osteogenic efficiency in bone tissue. Deteriorated cellular functions will exacerbate bone loss in PMOP patients [8]. This study found that silencing EPB41L4A-AS1 inhibited ADSC proliferation and migration while promoting apoptosis, indicating that EPB41L4A-AS1 improves bone formation by maintaining normal cellular functions of ADSCs. In the bone microenvironment, angiogenesis provides nutrients and oxygen for bone tissue. Extracellular matrix metabolism maintains the structural stability of bone tissue, and inflammation homeostasis prevents excessive damage to bone tissue. These three processes work together with ADSC osteogenic differentiation to maintain the balance of bone metabolism [32, 33]. Silencing EPB41L4A-AS1 was found to downregulate the mRNA expression of angiogenic factors, cause an imbalance in the expression of MMP-2 and TIMP-1, and increase the protein secretion of pro-inflammatory factors. This confirmed that EPB41L4A-AS1 could maintain the homeostasis of the bone microenvironment by regulating these major processes. It is hypothesized that EPB41L4A-AS1 may contribute to the maintenance of bone metabolic homeostasis by enhancing ADSC osteogenic differentiation, improving cellular function, and regulating bone microenvironment-associated processes in vitro. Whether these in vitro observations translate to systemic bone protective effects in vivo remains to be determined. EPB41L4A-AS1 shows unique regulatory features in PMOP compared to MALAT1, MIR155HG, and lncTIMP3 [23–25]. While MALAT1 promotes osteogenesis by inhibiting NEDD4-mediated RUNX1 degradation, MIR155HG suppresses it via miR-155-5p/DKK1, and lncTIMP3 enhances it through miR-214/Smad4—all acting downstream of osteogenic signaling but with opposing effects. In contrast, EPB41L4A-AS1: [1] coordinately regulates ADSC osteogenic differentiation, cellular function, and bone microenvironment homeostasis; [2] intersects inflammatory networks via miR-302d-3p–mediated ceRNA crosstalk—aligning with PMOP’s chronic low-grade inflammation; and [3] shows serum level correlation with fracture risk, supporting its potential role as diagnostic biomarker. The therapeutic implications of EPB41L4A-AS1 modulation remain speculative in the absence of in vivo efficacy data. However, its bone-specific cellular origin and direct links to estrogen signaling remain unclear and warrant further study. In the pathological context of PMOP, estrogen deficiency represents a central driver of ADSC dysfunction. Estrogen regulates the expression of osteogenic transcription factors including RUNX2 and SP7 through ERα/β signaling pathways, and its decline directly suppresses the osteogenic potential of ADSCs [34, 35]. Concurrently, estrogen loss elevates inflammatory cytokines (e.g., TNF-α, IL-6) and exacerbates oxidative stress within the bone marrow microenvironment, further compromising ADSC proliferation and viability [36, 37]. The downregulation of EPB41L4A-AS1 observed in this study likely constitutes a downstream manifestation of this pathological cascade, wherein its ceRNA-mediated derepression of miR-302d-3p amplifies ADSC dysfunction and establishes a self-perpetuating vicious cycle.

MiRNAs and lncRNAs commonly form ceRNA networks to jointly regulate the progression of bone diseases [38]. miR-302d-3p is involved in the regulation of inflammation, apoptosis, and extracellular matrix metabolism in OA and IDD, and its abnormal expression is closely linked to disease advancement [13–15]. It also regulates the pluripotency of stem cells and adipogenic differentiation of ADSCs [16, 39]. Bioinformatics analysis predicted a binding relationship between miR-302d-3p and EPB41L4A-AS1. This study showed that serum miR-302d-3p levels were elevated in PMOP patients, especially in those with fractures. During ADSC osteogenic differentiation, miR-302d-3p expression declined over time, inversely to EPB41L4A-AS1, indicating functional crosstalk in osteogenesis and PMOP development. RIP and dual-luciferase assay confirmed that EPB41L4A-AS1 directly targeted miR-302d-3p. Rescue experiments showed that co-inhibition of EPB41L4A-AS1 and miR-302d-3p reversed the inhibitory effect of EPB41L4A-AS1 silencing on osteogenic differentiation, cellular functions, and bone microenvironment. These results demonstrated that EPB41L4A-AS1 promotes ADSC osteogenic differentiation and maintains bone microenvironment homeostasis by inhibiting miR-302d-3p in vitro, thereby modulating osteogenic differentiation and associated cellular processes. These findings establish a mechanistic proof-of-concept rather than evidence of disease-modifying activity. Mechanistically, CUL3 was identified as a direct downstream target of miR-302d-3p. In patients with osteoporosis, CUL3 expression is reduced because it is negatively regulated by the lncRNA XIST. As a scaffold protein for E3 ubiquitin ligase, CUL3 plays a direct role in osteoblast differentiation by controlling the ubiquitination and degradation of Nrf2 [40]. It also regulates angiogenesis through pathways like VEGFR2/NOTCH1 and affects extracellular matrix remodeling and inflammatory signaling via the ubiquitin-proteasome system [41, 42]. Notably, it is the only gene that fulfills both the conditions of being downregulated in expression and having four distinct functional regulatory roles. The rescue-reversion experiments demonstrated that phenotypic restoration following miR-302d-3p inhibition was strictly dependent on CUL3 expression, establishing a hierarchical EPB41L4A-AS1/miR-302d-3p/CUL3 regulatory cascade. This closed-loop validation not only confirms the specificity of the RNA interference tools employed but also positions CUL3 as a pivotal molecular switch translating upstream non-coding RNA signals into functional outcomes in ADSCs. CUL3 functions as the core scaffold of the KEAP1-CUL3-RBX1 E3 ubiquitin ligase complex that targets Nrf2 for proteasomal degradation. In this study, Nrf2 protein levels inversely correlated with CUL3 expression across the rescue-reversion groups, providing a mechanistic rationale for the observed microenvironmental phenotypes: CUL3 knockdown-induced Nrf2 accumulation may disrupt redox homeostasis and indirectly perturb angiogenic factor expression, extracellular matrix remodeling, and inflammatory balance. While these data establish Nrf2 as a plausible downstream mediator linking CUL3 to bone microenvironment regulation, direct assessment of Nrf2 transcriptional activity and canonical target genes (e.g., HO-1, NQO1) was not performed; thus, this interpretation remains mechanistically inferred. Although this study identified the binding sequence between EPB41L4A-AS1 and miR-302d-3p, it did not assess the cross-species evolutionary conservation of this specific miRNA response element. Since MREs derived from lncRNAs typically evolve quickly and our findings are mainly based on functional validation in human cells, future research using animal models may provide further insight into the species conservation of this regulatory pathway. Elevated serum miR-302d-3p likely reflects increased local release from bone tissue. Under pathological conditions, osteocytes, osteoclasts, and bone marrow mesenchymal stem cells actively secrete exosomes containing miRNAs into circulation, providing a plausible route for miR-302d-3p entry into the bloodstream. Furthermore, circulating miR-302d-3p may be re-uptaken by bone-resident cells via exosomes or similar carriers, establishing a “local-to-circulation-to-local” feedback loop that persistently suppresses osteogenic differentiation and promotes inflammatory responses in target tissues, thereby exerting systemic regulatory effects during PMOP progression.

Recently, related research also identified EPB41L4A-AS1 as a diagnostic marker promoting osteogenic differentiation in periodontal ligament fibroblasts via the miR-214-3p/YAP1 axis [6]. However, this study differs in three key aspects: (1) disease context—systemic PMOP versus localized periodontitis; (2) cellular model—ADSCs with robust multilineage potential versus fibroblasts with limited differentiation capacity; and (3) mechanism—miR-302d-3p/CUL3-mediated bone microenvironment homeostasis versus miR-214-3p/YAP1 signaling. These convergent yet context-specific findings suggest EPB41L4A-AS1 serves as an adaptive master regulator of osteogenesis across distinct pathological settings. We extend this paradigm by establishing serum-based fracture risk prediction and elucidating comprehensive bone microenvironment modulation (angiogenesis, matrix remodeling, and inflammatory balance), thereby broadening the mechanistic understanding of EPB41L4A-AS1 in the context of systemic bone metabolic disorders. These insights are confined to in vitro mechanistic exploration and do not constitute evidence of in vivo translational potential.

The specificity of RNA interference tools is a critical concern in mechanistic studies. To address this, we employed a multi-layered validation strategy. First, negative controls (si-NC, mimic-NC, and inhibitor-NC) consistently showed no significant effects on target gene expression or cellular phenotypes, ruling out non-specific transfection artifacts. Second, the dual-luciferase reporter assay confirmed sequence-specific binding between EPB41L4A-AS1 and miR-302d-3p, as well as miR-302d-3p and CUL3, as the seed-region mutation completely abolished the regulatory interaction. Most importantly, we performed functional rescue-reversion experiments to establish a closed-loop validation: while miR-302d-3p inhibitor reversed the impaired osteogenesis caused by EPB41L4A-AS1 silencing, further knockdown of the downstream effector CUL3 under this rescued condition precisely reinstated the osteogenic defects, cellular dysfunction, and bone microenvironment imbalance. The si-NC(CUL3) control exhibited no such effects. This “silencing → rescue → reversion” paradigm, where phenotypic changes strictly follow the predicted regulatory hierarchy, provides strong functional evidence that si-EPB41L4A-AS1, inhibitor-miR-302d-3p, and si-CUL3 all act with high target specificity rather than through off-target mechanisms. The interference sequences used in this study are mature sequences previously validated in the literature. Although the functional rescue-reversion strategy and strict negative controls provide robust internal evidence for the specificity of our RNA interference tools, this study did not employ a second non-overlapping siRNA or overexpression rescue constructs to further rule out potential off-target effects. Future investigations will incorporate these complementary approaches to definitively confirm the sequence-specific actions of the targeted RNAs.

The present study is explicitly framed as a clinical biomarker and in vitro mechanistic proof-of-concept investigation. All claims regarding the EPB41L4A-AS1/miR-302d-3p/CUL3 axis are derived from serum correlation analyses and ADSC-based cellular experiments. Consequently, any inference about disease modification, therapeutic intervention, or in vivo bone microenvironment regulation should be interpreted with caution and regarded as hypothetical pending appropriate animal validation. This study has several limitations. First, we lack in vivo validation: an ovariectomized (OVX) mouse model is needed to test whether modulating EPB41L4A-AS1 or the EPB41L4A-AS1/miR-302d-3p/CUL3 axis improves bone mineral density, microarchitecture (µCT), and histomorphometry—essential steps to confirm its therapeutic potential for PMOP. However, our institution lacks SPF-grade animal facilities and µCT infrastructure; OVX model establishment and lncRNA modulation require 3–6 months and exceed current funding and timeline constraints. Second, although our in vitro data are robust—including a closed-loop silencing-rescue-reversion strategy, seed-mutant luciferase assays, and multi-dimensional bone microenvironment analysis—they remain mechanistic proof-of-concept and require in vivo confirmation before clinical translation can be claimed. Third, this study employed commercially available ADSCs from healthy premenopausal women aged 32–48 years. While this selection ensures standardized viability, batch consistency, and ethical feasibility, these cells possess fundamentally different baseline epigenetic and functional states compared with ADSCs residing in the estrogen-deficient, senescent adipose microenvironment characteristic of PMOP. Premenopausal ADSCs maintain robust endogenous estrogen signaling, which intrinsically elevates baseline osteogenic potential through ERα/β-mediated pathways and concurrently suppresses inflammatory tone and oxidative stress. Consequently, the rescue of osteogenic differentiation observed upon EPB41L4A-AS1 manipulation may reflect restoration of an inherently robust differentiation capacity rather than reversal of the pathological suppression typical of PMOP-derived cells. Furthermore, the inflammatory and angiogenic phenotypes reported herein warrant cautious interpretation. In PMOP adipose tissue, chronic low-grade inflammation and cellular senescence establish a senescence-associated secretory phenotype (SASP) characterized by elevated basal secretion of TNF-α, IL-6, and MMP-2, alongside compromised angiogenic factor production secondary to microvascular dysfunction. By contrast, the premenopausal ADSCs used in this study likely exhibited low basal inflammatory cytokine levels and intact angiogenic responsiveness. Thus, the detected shifts in inflammatory mediators and pro-angiogenic factors following lncRNA modulation represent deviations from a physiologically normal baseline rather than modulation of a pathologically activated state. These findings should therefore be regarded as mechanistic proof-of-concept within a healthy cellular context; validation using primary ADSCs or BMSCs from postmenopausal osteoporotic patients is essential to confirm the disease relevance of the EPB41L4A-AS1/miR-302d-3p/CUL3 axis.

## Conclusion

Low serum EPB41L4A-AS1 expression in PMOP patients might serve as a molecular marker for early diagnosis and fracture risk prediction. Cell experiments revealed that the EPB41L4A-AS1/miR-302d-3p/CUL3 axis regulated ADSC osteogenic differentiation and bone microenvironment-associated functions in vitro, providing a mechanistic proof-of-concept for understanding PMOP pathophysiology. These findings warrant further validation in in vivo models before any translational or therapeutic implications can be inferred.

## Supplementary Information

Supplementary file 1: Supplementary Figure 1. The effects of miR-302d-3p overexpression in osteogenic differentiation cell model on miR-302d-3p expression (A), the mRNA levels of osteogenic differentiation-related genes OPN, RUNX2, BMP-2, and COL1 (B), the ALP activity (C), the staining intensity of mineralized nodules (D), as well as the proliferative activity (E), apoptosis rate (F), and migration rate (G) of ADSCs. Data were analyzed using repeated‑measures ANOVA with Bonferroni post‑hoc pairwise comparisons. P values for each comparison are provided in the figure. Data are presented as mean ± SD. *, P < 0.05; **, P < 0.01; ***, P < 0.001.

Supplementary file 2: Supplementary Figure 2. The effects of miR-302d-3p overexpression in osteogenic differentiation cell model on the mRNA levels of pro-angiogenic factors VEGF, bFGF, ANG-1, and PDGF (A), the protein levels of extracellular matrix remodeling markers MMP-2 and TIMP-1 (B), and the protein levels of inflammatory factors TNF-α, IL-6, IL-10, and TGF-β1 (C). Data were analyzed using repeated‑measures ANOVA with Bonferroni post‑hoc pairwise comparisons. P values for each comparison are provided in the figure. Data are presented as mean ± SD. **, P < 0.01; ***, P < 0.001.

Supplementary file 3: Supplementary Figure 3. Stability of serum GAPDH Ct values across study cohorts. The mean Ct values of serum GAPDH (averaged from three technical replicates per sample) are shown for healthy controls (n = 118), PMOP-non-fracture patients (n = 75), and PMOP-fracture patients (n = 71). No statistically significant difference was observed among the three groups (one-way ANOVA: F = 0.256, P = 0.774). Data are presented as box plots.

Supplementary file 4.

Supplementary file 5.

Supplementary file 6.

Supplementary file 7.

## Data Availability

No datasets were generated or analysed during the current study.
