# Supplementary material for: LncRNA EPB41L4A-AS1 regulates osteogenic differentiation and bone microenvironment-associated functions in ADSCs by targeting miR-302d-3p
Source: Hereditas. 2026 Aug 11;163:127. doi: 10.1186/s41065-026-00721-7 (PMC13746403; doi:10.1186/s41065-026-00721-7)
Supplement: Supplementary file 1 [file 41065_2026_721_MOESM4_ESM.docx]

**Supplementary Table 1** Primers used in this study

| Gene Name | Primer Sequence (5′-3′) | |
| --- | --- | --- |
| LncRNA EPB41L4A-AS1 | Forward | CCACCCTGAGTCTGGTGAGT |
|  | Reverse | CCTGGCATAGTCGATGATGTA |
| miR-302d-3p | Forward | AGTGCTTCCATGTTTGAGTGT |
|  | Reverse | AGTGCAGGGTCCGAGGTATT |
| GAPDH | Forward | GAAGGTGAAGGTCGGAGTC |
|  | Reverse | GAAGATGGTGATGGGATTTC |
| U6 | Forward | CTCGCTTCGGCAGCACA |
|  | Reverse | AACGCTTCACGAATTTGCGT |
| RUNX2 | Forward | CGCCTCACAAACAACCACAG |
|  | Reverse | ACTGCTTGCAGCCTTAAATGAC |
| BMP2 | Forward | GCGTGAAAAGAGAGACTGCG |
|  | Reverse | ACCATGGTCGACCTTTAGGAG |
| OPN | Forward | AACGCCGACCAAGGAAAACT |
|  | Reverse | GCACAGGTGATGCCTAGGAG |
| COL1 | Forward | ACGAAGACATCCCACCAATC |
|  | Reverse | AGATCACGTCATCGCACAAC |
| VEGF | Forward | AGGCAGAATCATCACGAAGT |
|  | Reverse | AGGGTCTCGATTGGATGGCA |
| bFGF | Forward | CCCATTCCTTGGTTCATCGT |
|  | Reverse | CCATAGCCTCCGCAACATAC |
| ANG-1 | Forward | TGGGGGAGGTTGGACTGTAA |
|  | Reverse | GCCTCTGACTGGTAATGGCA |
| PDGF | Forward | CCCACCCTCTAGCTTCGTTG |
|  | Reverse | GGCTCGGGTCAGTCTGTCTA |
| CUL3 | Forward | TGTGGAGAACGTCTACAATTTGG |
|  | Reverse | GCGCCTCTGTCTACGACTT |
