# Supplementary material for: LncRNA EPB41L4A-AS1 regulates osteogenic differentiation and bone microenvironment-associated functions in ADSCs by targeting miR-302d-3p
Source: Hereditas. 2026 Aug 11;163:127. doi: 10.1186/s41065-026-00721-7 (PMC13746403; doi:10.1186/s41065-026-00721-7)
Supplement: Supplementary file 2 [file 41065_2026_721_MOESM5_ESM.docx]

**Supplementary Table 2** Diagnostic value of β-CTX, OCN, T scores and combined models

| Parameters | AUC | 95% CI | Sensitivity (%) | Specificity (%) | *P* value |
| --- | --- | --- | --- | --- | --- |
| β-CTX | 0.677 | 0.612 to 0.743 | 65.07 | 70.34 | <0.001 |
| OCN | 0.640 | 0.573 to 0.708 | 60.27 | 67.80 | <0.001 |
| T scores | 0.733 | 0.671 to 0.795 | 71.23 | 75.42 | <0.001 |
| Combined | 0.913 | 0.880 to 0.946 | 74.66 | 89.83 | <0.001 |

Abbreviations: β-CTX, β-isomerized C-terminal telopeptide of type I collagen; OCN, osteocalcin; AUC, the area under curve; 95% CI, confidence interval.
