# Supplementary material for: LncRNA EPB41L4A-AS1 regulates osteogenic differentiation and bone microenvironment-associated functions in ADSCs by targeting miR-302d-3p
Source: Hereditas. 2026 Aug 11;163:127. doi: 10.1186/s41065-026-00721-7 (PMC13746403; doi:10.1186/s41065-026-00721-7)
Supplement: Supplementary file 3 [file 41065_2026_721_MOESM7_ESM.docx]

**Supplementary attachment 1** The protein levels of MMP-2, TIMP-1, and Nrf2 in different treatment groups
